# Gene polymorphisms associated with corneal curvature, astigmatism and its vector components in children

**DOI:** 10.1186/s40662-025-00464-y

**Published:** 2025-12-01

**Authors:** Ebenezer Zaabaar, Erica Shing, Yu Yao Wang, Ka Wai Kam, Pancy O. S. Tam, Alvin L. Young, Clement C. Tham, Chi Pui Pang, Jason C. Yam, Li Jia Chen

**Affiliations:** 1 Department of Ophthalmology and Visual Sciences, The Chinese University of Hong Kong, Hong Kong, China; 2 Department of Ophthalmology and Visual Sciences, Prince of Wales Hospital, Hong Kong, China; 3 Hong Kong Eye Hospital, Hong Kong, China; 4 Hong Kong Hub of Paediatric Excellence, The Chinese University of Hong Kong, Hong Kong, China; 5 Shantou University/The Chinese University of Hong Kong Joint Shantou International Eye Centre, Shantou, China

**Keywords:** Corneal curvature, Astigmatism, Vector components, Children, Gene polymorphism

## Abstract

**Background:**

While parental astigmatism is a known risk factor for childhood astigmatism, the molecular genetic basis remains elusive. Previous genetic studies, largely confined to adult corneal and refractive astigmatism (CA and RA), did not address internal astigmatism (IA) and astigmatism vector components. We aimed to determine whether genes previously identified to have associations with corneal curvature (CR), CA, and RA in adults similarly occur for CR, CA, RA, IA, and astigmatism vector components (J0 and J45) in children.

**Methods:**

Fourteen polymorphisms in nine loci were genotyped in 2167 Chinese children. Linear and logistic regression analyses were conducted to evaluate the association of the polymorphisms with CR, CA, RA, IA, and astigmatism vector components, which were determined by keratometry, cycloplegic refraction, or Fourier transformation.

**Results:**

*FMNL2* rs1579050 was associated with CA (additive: β = 0.158, *P* = 0.0028; dominant: β = 0.163, *P* = 0.0034), J0_(CA)_ (additive: β = 0.081, *P* = 0.0031), and an increased risk of dichotomous RA (additive: OR = 1.609, *P* = 0.0028; dominant: OR = 1.671, *P* = 0.0020), whereas *NHSL1* rs4896367 was associated with J0_(IA)_ (recessive: β = 0.058, *P* = 0.0002) and a lower risk of dichotomous IA (recessive: OR = 0.577, *P* = 0.0007). *PDGFRA* rs2228230 was also associated with J0_(IA)_ (dominant: β = −0.034, *P* = 0.0012). The predisposition to CA and RA increased with the risk alleles of *FMNL2* rs1579050.

**Conclusions:**

These findings reveal genetic contributions to childhood astigmatism and demonstrate that vector-based decomposition may facilitate more precise mapping of its genetic determinants.

**Supplementary Information:**

The online version contains supplementary material available at 10.1186/s40662-025-00464-y.

## Background

Astigmatism is a prevalent refractive error resulting from meridional asymmetry in the curvature of the cornea (CR) and/or lens of the eye [1]. This asymmetry causes an uneven refraction of light rays to different points on the retina, rather than to a single point, leading to distorted and blurred vision. If left uncorrected, astigmatism can result in amblyopia [2], the most common cause of monocular vision loss in children [3, 4]. Visual deficits associated with the condition have been shown to adversely affect cognitive functions, language development, and fine motor skills in children [5]. Total or refractive astigmatism (RA) predominantly arises from corneal astigmatism (CA), with a lesser contribution from internal astigmatism (IA), which is largely of lenticular origin [6]. The global pooled prevalence of astigmatism has been estimated to be 40.4% in adults and 14.9% in children, though regional variations exist [7]. In Hong Kong, the prevalence in children is even higher, affecting up to 34.7% of 6–8-year-olds for RA and 64.7% for CA [8–10]. Interestingly, both the prevalence and severity of RA and CA increased significantly in Hong Kong Chinese children following the COVID-19 pandemic [10]. The rate of RA ≥ 1.0 diopter (D) rose from 21.4% in 2015 to 34.7% in 2022–2023, while CA ≥ 1.0 D increased from 59.8% to 64.7% over the same period.

Questions about the causes of astigmatism persist, with existing evidence showing several key contributing factors, including age, extraocular muscle tension, visual feedback, eyelid pressure, environmental influences, and genetic factors [10–12]. The support for a genetic component is considerable, with initial clues coming from epidemiology, which shows variations in rates of astigmatism across different ethnic populations [8, 9, 13–15]. Adding to this, family studies suggest that individuals with siblings or parents who have astigmatism are at a greater risk [16]. In Hong Kong, parental astigmatism was associated with a dose-dependent increase in the likelihood of astigmatism in children [8]. The genetic contribution has been quantified by heritability estimates approaching 50% for CA and RA [17]. Most conclusively, genome-wide association studies (GWASs) have identified single-nucleotide polymorphisms (SNPs) associated with the condition [18–22].

Despite the compelling evidence for a heritable component, several methodological challenges and gaps remain in astigmatism genetics. A key limitation is the predominant focus of genetic studies on adults [18–22], despite evidence that refractive error genetic effects may vary across ages [23–25]. Furthermore, analytical approaches may obscure genetic associations. Specifically, analysing astigmatism as a scalar rather than a vector quantity results in information loss and cancellation artifacts, where identical cylinder magnitudes with orthogonal axes would be indistinguishable though they may have fundamentally different biological underpinnings. Power vector analysis offers a more robust framework for analyzing the magnitude and axis of astigmatism simultaneously [26, 27]. Additionally, twin and heritability studies suggest that nonadditive genetic factors explain most of the variance in CR and astigmatism [23, 28], but association studies frequently rely solely on additive models, potentially missing recessive or dominant effects. Hammond et al. found that nonadditive genetic factors accounted for 42%–61% of CA variance, while additive genetic factors accounted for 4%–18% [23]. The corresponding proportions for RA variance were 47%–49% and 1%–4%, respectively [23]. Moreover, the genetics of IA remain understudied. To address these gaps, we selected SNPs previously identified to be associated with CR, CA, and RA in adults, aiming to investigate their associations with flat and steep corneal radii of curvature (K1 and K2, respectively), mean corneal radius of curvature (CR), CA, RA, IA, and the vector components of astigmatism in children, using additive and nonadditive genetic models.

## Methods

### Study design and participants

This is a cross-sectional study that included 2167 unrelated Hong Kong Chinese children aged 4–11 years, as part of the Hong Kong Children’s Eye Study [29, 30]. The age range of 4–11 years was chosen for the following reasons. First, the human cornea reaches its adult dimensions by age 2 [31, 32]; thus, our lower limit of 4 years ensures that the corneal measurements reflect a post-growth, stable state. Second, by age 4, most children have the cognitive and cooperative capacity necessary to undergo reliable ophthalmic examinations, ensuring the collection of valid and accurate data. Younger children may struggle to follow instructions or remain still during assessments, which can compromise the quality of the results. Finally, the upper limit of 11 years allows us to specifically investigate childhood astigmatism before the onset of adolescence, thereby minimizing the potential confounding effects of significant hormonal changes on ocular development. All procedures were performed in compliance with relevant laws and institutional guidelines and have been approved by the Ethics Committee of the Chinese University of Hong Kong (Research Ethics Committee Reference Number: 2015.033). The study adhered to the tenets of the World Medical Association Declaration of Helsinki. The privacy rights of the subjects were observed, and written informed consent was obtained from parents or legal guardians, along with verbal consent from each child participant. Subjects with ophthalmic conditions that could affect refraction, such as keratoconus or ocular syndromes, were excluded from the study. Participants with signs of keratoconus were excluded based on specific diagnostic criteria, including the presence of a typical keratoconus pattern on corneal topography, characterized by localised steepening or an asymmetric bowtie configuration, with the steep keratometry reading exceeding 47 D. Additionally, any observed clinical signs of keratoconus (corneal thinning, scarring, apical protrusion, Fleischer’s ring, or Vogt’s striae) or history of corneal surgery led to exclusion.

### Phenotypic assessments

Each participant underwent a comprehensive ophthalmic examination that included, but not limited to, visual acuity assessment, cycloplegic refraction, keratometry, slit-lamp examination, and fundoscopy. Cycloplegia was achieved with cycloplegic eye drops, including cyclopentolate 1% (Cyclogyl, Alcon-Convreur, Rijksweg, Belgium) and tropicamide 1% (Santen, Osaka, Japan). The cycloplegic procedure involved administering at least two cycles of eye drops. Each cycle consisted of one drop of 1% cyclopentolate and one drop of 1% tropicamide, applied to both eyes 5 min apart. A second cycle was given 10 min after the first. If the pupil size was less than 6.0 mm or a light reflex persisted 30 min after the second cycle, a third cycle was administered. Additional cycles were used as needed to achieve full cycloplegia. Refraction was measured 30 min after the final drop. Five readings were taken and averaged, but only if all readings were within 0.25 D of each other. Children who did not achieve this level of cycloplegia or could not complete the procedure were excluded from the study. Cycloplegic refraction was conducted using an auto refractometer (Nidek ARK-510A, Gamagori, Japan). Corneal parameters, including K1 and K2 were measured with noncontact partial-coherence laser interferometry (IOL Master; Carl Zeiss Meditec, Orberkochen, Germany), and the average (CR) of the K1 and K2 was determined. The negative cylinder component of the cycloplegic refraction was used as RA, and CA was calculated as the absolute difference between K1 and K2. RA and CA were decomposed into their vector components (J0 and J45) using Fourier transformation, as described by the following equations [33]: J0_(RA)_ = −C/2 × Cos 2ɑ_RA_; J45_(RA)_ = −C/2 × Sin 2ɑ_RA_; J0_(CA)_ = C/2 × Cos 2ɑ_CA_; J45_(CA)_ = C/2 × Sin 2ɑ_CA_. These further enabled the calculation of IA and its vector components using the following formulae: J0_(IA)_ = J0_(RA)_ − J0_(CA)_; J45_(IA)_ = J45_(RA)_ − J45_(CA)_; IA = −2 √[J0_(IA)_^2^ + J45_(IA)_^2^]. Vector component calculations required measurements from a single eye. Thus, we selected the right eye for all analyses, as measurements for K1, K2, CA, and RA were highly correlated between eyes (*r* = 0.97, 0.96, 0.79, and 0.81, respectively, all *P* < 0.001).

Positive values of J0 indicate with-the-rule (WTR) astigmatism, while negative values indicate against-the-rule (ATR) astigmatism [34]. J45 represents oblique astigmatism with a cross-cylinder set at 45° and 135°. Negative J45 values suggest astigmatism around 45°, and positive values indicate astigmatism around 135° [34].

### SNPs selection and genotyping

We conducted a meta-analysis of genetic associations for RA, CA, and CR [17], and selected fourteen SNPs near/at nine candidate genes/loci with pooled *P* values of 1 × 10^−5^ or lower and a minor allele frequency greater than 5% in Southern Chinese (CHS), as per the 1000 Genomes Project Phase 3 allele frequencies (Ensembl release 113/Ensembl Genomes 60: East Asian population subset). The selected SNPs included *RPS6KC1* rs12144639, *ZC3H11B* rs7525202, *FMNL2* rs1579050, *TRAF3IP1* rs77008212, *PDGFRA* rs17084051, rs2114039 and rs2228230, *BMP3* rs1353386, *CASC15* rs4712652 and rs10946507, *NHSL1* rs4896367 and rs4620141, and *FBN1* rs9806595 and rs25458. DNA was extracted from whole blood or oral mucosa samples using the QIAamp DNA Blood kit (Qiagen, Hilden, Germany). A description of the selected SNPs is provided in Supplementary Table 1. All SNPs were genotyped using TaqMan SNP assays (Applied Biosystems, Foster City, CA) with a Roche LightCycler 480 Real-Time PCR System (Roche, Basel, Switzerland).

### Statistical analyses

Data analysis was carried out using PLINK (v1.9) and R (v.4.4.3, R Foundation). A quality control check was implemented to eliminate SNPs that did not meet Hardy–Weinberg equilibrium (HWE; *P* < 0.05, Supplementary Table 2). Individuals with more than 10% missing genotype data were excluded. Additionally, variants missing in more than 10% of individuals were removed. This is because these samples were likely to have been generated from low-quality DNA and could also have higher-than-average genotyping error rates. Besides, statistical guidance studies have indicated that analyses may generate biased results with more than 10% missingness [35, 36]. The allelic associations of each of the 14 SNPs with the phenotypes were assessed using linear regression while controlling for age and sex. Additive, dominant, and recessive genetic models were employed in the analyses. The analysis for each model was run separately, and the analysis for each astigmatism or CR component was also conducted separately. A *P* value of <0.05 was considered nominally significant, and to account for multiple comparisons, we applied the Bonferroni correction, establishing a threshold of <0.00357 (0.05/14) to indicate study-wide significance for each separate analysis. To help control the expected proportion of false positives among the rejected hypotheses, we also applied the Benjamini–Hochberg false discovery rate (FDR) approach for multiple testing adjustment, with the FDR *P* value threshold = $\left( {\frac{{{\text{rank}}\,{\text{of}}\,P\;{\text{value}} \times 0.05}}{14}} \right)$. Furthermore, the effects of the interactions of the SNPs with sex and age were evaluated. We assessed the relationship between the SNPs and astigmatism as a binary or dichotomous outcome [cylinder power (RA and IA) ≤ −0.75 D; cylinder power (CA) ≥ 0.75 D], using logistic regression adjusted for age and sex. Moreover, the Cochran–Armitage trend test was conducted to identify if the likelihood of being astigmatic increased with the number of risk alleles. The analysis was also stratified by age (4–7 years and 8–11 years) and sex.

## Results

The study comprised 1176 (54%) boys and 991 (46%) girls, with a mean age of 7.67 ± 1.05 years. Table 1 presents the demographic and clinical characteristics of the study participants. There was a strong correlation between each pair of K1, K2 and CR (K1 and K2: *r* = 0.89, *P* < 0.001; K1 and CR: *r* = 0.97, *P* < 0.001; K2 and CR: *r* = 0.97, *P* < 0.001), as well as between each type of astigmatism and its J0 (CA and J0_(CA)_: *r* = 0.99, *P* < 0.001; RA and J0_(RA)_: *r* = −0.88, *P* < 0.001; IA and J0_(IA)_: *r* = 0.93, *P* < 0.001), while the remaining pairs of parameters demonstrated moderate to weak correlations (Supplementary Figure 1). Age was weakly correlated with all the phenotypes (Supplementary Figure 1). The vector analysis showed a positive mean J0 (WTR astigmatism) for CA and RA but a negative mean J0 (ATR astigmatism) for IA. On the other hand, mean J45 was negative (astigmatism around 45°) for CA and RA and positive (astigmatism around 135°) for IA.

**Table 1:** Baseline characteristics of study subjects

Parameter	Value
Total number, N	2167
Age (years)	7.67 ± 1.05
Age group, n (%)	
4–7 years	1010 (46.6)
8–11 years	1157 (53.4)
Sex, n (%)	
Male	1176 (54)
Female	991 (46)
Flat keratometry reading (K1)	42.93 ± 1.36 D
Steep keratometry reading (K2)	44.13 ± 1.54 D
Mean keratometry reading (CR)	43.53 ± 1.41 D
Corneal astigmatism (CA)	1.21 ± 0.67 D
J0_(CA)_	0.58 ± 0.34 D
J45_(CA)_	−0.06 ± 0.16 D
Refractive astigmatism (RA)	−0.68 ± 0.67 D
J0_(RA)_	0.28 ± 0.35 D
J45_(RA)_	−0.03 ± 0.15 D
Internal astigmatism (IA)	−0.69 ± 0.38 D
J0_(IA)_	−0.30 ± 0.21 D
J45_(IA)_	0.03 ± 0.13 D

*SNP FMNL2* rs1579050 yielded significant associations (both Bonferroni and FDR corrected) with CA (additive: β = 0.158, *P* = 0.0028; dominant: β = 0.163, *P* = 0.0034; recessive: β = 0.324, *P* = 0.2823, Table 2), J0_(CA)_ (additive: β = 0.081, *P* = 0.0031; dominant: β = 0.083, *P* = 0.0038; recessive: β = 0.169, *P* = 0.2694, Table 2), and dichotomous RA (additive: OR = 1.609, *P* = 0.0028; dominant: OR = 1.671, *P* = 0.0020; recessive: OR = 1.283, *P* = 0.7857, Table 3), whereas *NHSL1* rs4896367 demonstrated significant associations (both Bonferroni and FDR corrected) with J0_(IA)_ (additive: β = 0.018, *P* = 0.0089; dominant: β = 0.011, *P* = 0.2275; recessive: β = 0.058, *P* = 0.0002, Table 4) and dichotomous IA (additive: OR = 0.842, *P* = 0.0124; dominant: OR = 0.894, *P* = 0.2136; recessive: OR = 0.577, *P* = 0.0007, Table 4). The association of *PDGFRA* rs2228230 with J0_(IA)_ was also significant (both Bonferroni and FDR corrected; additive: β = −0.026, *P* = 0.0058; dominant: β = −0.034, *P* = 0.0012; recessive: β = 0.017, *P* = 0.6203, Table 4). The Cochran–Armitage trend test showed a significant association of *FMNL2* rs1579050 with CA (*P* = 0.0034, Supplementary Table 3) and RA (*P* = 0.0023, Supplementary Table 3), revealing that the risk of developing CA and RA increased with the number of risk alleles, indicating a significant dose–response or linear relationship. There was also a significant trend of the protective effect of *NHSL1* rs4896367 on IA, but at a nominal level (*P* = 0.0110, Supplementary Table 3). SNPs near other loci, including *RPS6KC1* rs12144639, *ZC3H11B* rs7525202, *TRAF3IP1* rs77008212, and *CASC15* rs4712652, were nominally associated with various types and components of astigmatism, as well as CR (Tables 2, 3, 4).

**Table 2:** Association of SNPs with corneal curvature and corneal astigmatism

CHR	Locus	SNP	Model	EA	K1		K2		CR		CA		J0_(CA)_		J45_(CA)_	
					β	*P*	β	*P*	β	*P*	β	*P*	β	*P*	β	*P*
1	*RPS6KC1*	rs12144639	ADD	A	−0.015	0.7578	−0.041	0.4562	−0.028	0.5780	−0.019	0.4470	−0.010	0.4111	−0.002	0.7508
			DOM		−0.014	0.8135	−0.032	0.6316	−0.023	0.7069	−0.014	0.6371	−0.006	0.6769	−0.001	0.8634
			REC		−0.041	0.7589	−0.146	0.3419	−0.094	0.5041	−0.067	0.3280	−0.046	0.1908	−0.008	0.6315
1	*ZC3H11B*	rs7525202	ADD	G	0.059	0.1949	0.083	0.1061	0.071	0.1314	0.025	0.2841	0.003	0.8300	0.001	0.7920
			DOM		0.117	0.0400*	0.159	0.0140*	0.138	0.0200*	0.045	0.1212	0.016	0.2855	0.004	0.5513
			REC		−0.090	0.4141	−0.102	0.4133	−0.096	0.4004	−0.022	0.6950	−0.043	0.1267	−0.007	0.6133
2	*FMNL2*	rs1579050	ADD	G	−0.168	0.1086	0.000	0.9986	−0.084	0.4395	0.158	0.0028**	0.081	0.0031**	−0.011	0.3859
			DOM		−0.144	0.1890	0.029	0.8179	−0.058	0.6130	0.163	0.0034**	0.083	0.0038*	−0.010	0.4573
			REC		−1.181	0.0460*	−0.849	0.2082	−1.015	0.0990	0.324	0.2823	0.169	0.2694	−0.063	0.3741
2	*TRAF3IP1*	rs77008212	ADD	G	0.068	0.4746	0.112	0.3035	0.090	0.3642	0.050	0.2990	0.030	0.2211	0.003	0.8075
			DOM		0.073	0.4485	0.119	0.2765	0.096	0.3369	0.053	0.2771	0.032	0.2046	0.003	0.8187
			REC		−0.687	0.6041	−1.071	0.4770	−0.879	0.5229	−0.392	0.5608	−0.168	0.6232	0.035	0.8227
4	*PDGFRA*	rs17084051	ADD	A	0.064	0.2022	0.102	0.0750	0.083	0.1123	0.029	0.2612	0.009	0.4715	−0.007	0.2665
			DOM		0.075	0.2079	0.126	0.0620	0.100	0.1038	0.040	0.1835	0.014	0.3650	−0.006	0.3679
			REC		0.087	0.5446	0.096	0.5559	0.091	0.5392	0.000	0.9974	−0.005	0.8976	−0.017	0.3159
4	*PDGFRA*	rs2114039	ADD	C	0.073	0.0970	0.105	0.0370*	0.089	0.0520	0.026	0.2405	0.008	0.4743	−0.005	0.3232
			DOM		0.110	0.0530	0.159	0.0140*	0.134	0.0230*	0.043	0.1337	0.016	0.2874	−0.006	0.3834
			REC		0.038	0.7159	0.048	0.6845	0.043	0.6910	0.001	0.9977	−0.007	0.7894	−0.009	0.4714
4	*PDGFRA*	rs2228230	ADD	T	0.110	0.0610	0.138	0.0380*	0.124	0.0410*	0.036	0.2252	0.019	0.2120	−0.006	0.3945
			DOM		0.150	0.0210*	0.189	0.0110*	0.170	0.0120*	0.049	0.1424	0.026	0.1220	−0.005	0.5536
			REC		−0.142	0.4976	−0.171	0.4717	−0.156	0.4713	−0.038	0.7172	−0.027	0.6207	−0.029	0.2510
4	*BMP3*	rs1353386	ADD	A	0.024	0.6387	0.022	0.7067	0.023	0.6658	−0.003	0.8944	0.001	0.9536	−0.005	0.4387
			DOM		0.055	0.3533	0.051	0.4525	0.053	0.3909	−0.005	0.8752	0.001	0.9299	−0.005	0.4728
			REC		−0.136	0.3530	−0.127	0.4460	−0.132	0.3875	0.000	0.9983	−0.002	0.9617	−0.008	0.6334
6	CASC15	rs4712652	ADD	G	−0.062	0.3392	−0.022	0.7615	−0.042	0.5313	0.029	0.3723	0.006	0.7226	0.010	0.2007
			DOM		−0.073	0.2875	−0.045	0.5662	−0.059	0.4089	0.017	0.6222	−0.001	0.9341	0.013	0.1171
			REC		0.063	0.8360	0.385	0.2674	0.224	0.4797	0.313	0.0430*	0.160	0.0426*	−0.033	0.3667
6	CASC15	rs10946507	ADD	A	0.073	0.2331	0.048	0.4901	0.060	0.3413	−0.019	0.5500	−0.011	0.4714	−0.007	0.3605
			DOM		0.090	0.1777	0.068	0.3660	0.079	0.2528	−0.013	0.6987	−0.010	0.5510	−0.008	0.3449
			REC		−0.042	0.8635	−0.153	0.5805	−0.097	0.7000	−0.120	0.3326	−0.044	0.4873	−0.006	0.8487
6	*NHSL1*	rs4896367	ADD	C	−0.031	0.4727	−0.056	0.2535	−0.043	0.3317	−0.015	0.4880	−0.015	0.1967	0.001	0.7940
			DOM		−0.053	0.3496	−0.055	0.3990	−0.054	0.3619	0.007	0.8071	−0.002	0.9097	0.003	0.6476
			REC		−0.002	0.9817	−0.119	0.2684	−0.061	0.5373	−0.093	0.0530	−0.065	0.0081*	−0.002	0.8523
6	*NHSL1*	rs4620141	ADD	C	−0.009	0.8195	−0.042	0.3717	−0.025	0.5493	−0.022	0.2949	−0.008	0.4610	−0.004	0.4397
			DOM		−0.026	0.6744	−0.050	0.4706	−0.038	0.5504	−0.012	0.7039	0.001	0.9599	−0.003	0.6396
			REC		0.007	0.9269	−0.061	0.4616	−0.027	0.7197	−0.052	0.1568	−0.026	0.1693	−0.007	0.4110
15	*FBN1*	rs9806595	ADD	C	−0.060	0.1649	−0.070	0.1558	−0.065	0.1484	0.014	0.5119	0.000	0.9872	−0.001	0.8893
			DOM		−0.090	0.1140	−0.105	0.1044	−0.098	0.0990	0.001	0.9692	−0.004	0.8132	0.000	0.9979
			REC		−0.039	0.6794	−0.044	0.6834	−0.042	0.6729	0.067	0.1689	0.009	0.7198	−0.003	0.7636
15	*FBN1*	rs25458	ADD	G	−0.044	0.3085	−0.059	0.2308	−0.052	0.2518	0.009	0.6761	−0.002	0.8305	−0.007	0.1976
			DOM		−0.041	0.4734	−0.064	0.3240	−0.052	0.3762	−0.007	0.8144	−0.008	0.5972	−0.004	0.5687
			REC		−0.098	0.3001	−0.106	0.3257	−0.102	0.2998	0.063	0.1927	0.010	0.6843	−0.021	0.0620

*CHR* = chromosome; *SNP* = single nucleotide polymorphism; *EA* = effect allele; *K1* = flat keratometry reading; *K2* = steep keratometry reading; *CR* = mean keratometry reading; *CA* = corneal astigmatism; *J0*_*(CA)*_ = J0 of corneal astigmatism; *J45*_*(CA)*_ = J45 of corneal astigmatism; *ADD* = additive genetic model; *DOM* = dominant genetic model; *REC* = recessive genetic model

*P* values in bold with ** indicate study-wide significance level; * nominal significance level

**Table 3:** Association of SNPs with dichotomous astigmatism

CHR	Locus	SNP	Model	EA	CA		RA		IA	
					OR	*P*	OR	*P*	OR	*P*
1	*RPS6KC1*	rs12144639	ADD	A	1.080	0.3836	0.931	0.3581	1.045	0.5689
			DOM		1.127	0.2529	0.937	0.4848	1.000	0.9991
			REC		0.947	0.8170	0.817	0.3556	1.387	0.1165
1	*ZC3H11B*	rs7525202	ADD	G	0.943	0.4664	1.088	0.2356	1.074	0.3189
			DOM		0.909	0.3441	1.146	0.1296	1.039	0.6734
			REC		1.012	0.9522	0.992	0.9627	1.307	0.1120
2	*FMNL2*	rs1579050	ADD	G	1.922	0.0040*	1.609	0.0028**	1.256	0.1591
			DOM		1.912	0.0051*	1.671	0.0020**	1.252	0.1867
			REC		NA	NA	1.283	0.7857	2.133	0.4102
2	*TRAF3IP1*	rs77008212	ADD	G	1.174	0.3615	1.063	0.6836	1.330	0.0520
			DOM		1.168	0.3784	1.072	0.6455	1.349	0.0429*
			REC		NA	NA	NA	NA	NA	NA
4	*PDGFRA*	rs17084051	ADD	A	1.081	0.3866	1.024	0.7624	1.124	0.1375
			DOM		1.041	0.7019	1.059	0.5391	1.154	0.1246
			REC		1.558	0.1236	0.867	0.5371	1.128	0.5889
4	*PDGFRA*	rs2114039	ADD	C	1.111	0.1877	0.983	0.8035	1.103	0.1596
			DOM		1.111	0.2984	1.069	0.4566	1.150	0.1207
			REC		1.258	0.2368	0.719	0.0544	1.078	0.6446
4	*PDGFRA*	rs2228230	ADD	T	1.051	0.6365	0.952	0.5940	1.136	0.1635
			DOM		1.050	0.6742	0.939	0.5460	1.157	0.1536
			REC		1.140	0.7318	1.009	0.9787	1.143	0.6838
4	*BMP3*	rs1353386	ADD	A	1.082	0.3880	0.992	0.9198	0.944	0.4715
			DOM		1.007	0.9493	0.982	0.8499	0.905	0.2858
			REC		2.128	0.0211*	1.041	0.8609	1.129	0.5920
6	CASC15	rs4712652	ADD	G	1.090	0.4607	1.061	0.5586	1.003	0.9795
			DOM		1.061	0.6334	0.994	0.9552	1.019	0.8647
			REC		2.669	0.1903	3.981	0.0054*	0.732	0.5311
6	CASC15	rs10946507	ADD	A	1.091	0.4300	0.966	0.7193	1.042	0.6711
			DOM		1.122	0.3369	0.946	0.5985	1.050	0.6415
			REC		0.860	0.7168	1.203	0.6237	0.996	0.9918
6	*NHSL1*	rs4896367	ADD	C	0.871	0.0689	1.042	0.5433	0.842	0.0124*
			DOM		0.900	0.2989	1.078	0.4048	0.894	0.2136
			REC		0.700	0.0236*	0.993	0.9598	0.577	0.0007**
6	*NHSL1*	rs4620141	ADD	C	0.980	0.7845	1.074	0.2661	0.999	0.9917
			DOM		1.028	0.7961	1.066	0.5123	0.983	0.8618
			REC		0.904	0.4270	1.147	0.2282	1.022	0.8511
15	*FBN1*	rs9806595	ADD	C	1.016	0.8358	0.896	0.1115	0.980	0.7641
			DOM		0.998	0.9815	0.846	0.0629	0.938	0.4775
			REC		1.089	0.6212	0.943	0.6959	1.081	0.6002
15	*FBN1*	rs25458	ADD	G	1.005	0.9477	0.923	0.2415	0.924	0.2471
			DOM		1.003	0.9783	0.897	0.2271	0.885	0.1753
			REC		1.017	0.9218	0.919	0.5769	0.958	0.7764

*CHR* = chromosome; *SNP* = single nucleotide polymorphism; *EA* = effect allele; *CA* = corneal astigmatism; *RA* = refractive astigmatism; *IA* = internal astigmatism; *OR* = odds ratio; *ADD* = additive genetic model; *DOM* = dominant genetic model; *REC* = recessive genetic model

*P* values in bold with ** indicate study-wide significance level; * nominal significance level

**Table 4:** Association of SNPs with refractive and internal astigmatism

CHR	Locus	SNP	Model	EA	RA		J0_(RA)_		J45_(RA)_		IA		J0_(IA)_		J45_(IA)_	
					β	*P*	β	*P*	β	*P*	β	*P*	β	*P*	β	*P*
1	*RPS6KC1*	rs12144639	ADD	A	0.050	0.0435*	−0.018	0.1701	0.003	0.6140	−0.008	0.5614	−0.010	0.1875	0.006	0.2291
			DOM		0.048	0.1057	−0.016	0.3160	0.004	0.5714	−0.007	0.6662	−0.011	0.2425	0.006	0.2832
			REC		0.125	0.0660	−0.053	0.1408	0.001	0.9306	−0.024	0.5434	−0.021	0.3466	0.012	0.3989
1	*ZC3H11B*	rs7525202	ADD	G	−0.007	0.7591	0.001	0.9401	0.003	0.5911	−0.015	0.2435	−0.004	0.5921	0.001	0.8414
			DOM		−0.036	0.2094	0.014	0.3475	0.005	0.4714	−0.007	0.6548	−0.004	0.6760	0.000	0.9775
			REC		0.093	0.0950	−0.047	0.1044	−0.001	0.9316	−0.061	0.0510	−0.009	0.6235	0.005	0.6687
2	*FMNL2*	rs1579050	ADD	G	−0.111	0.0358*	0.076	0.0061*	0.000	0.9903	−0.017	0.5822	0.000	0.9934	0.010	0.3349
			DOM		−0.115	0.0380*	0.078	0.0071*	0.001	0.9573	−0.018	0.5792	0.001	0.9709	0.010	0.3899
			REC		−0.201	0.5027	0.154	0.3294	−0.015	0.8253	−0.020	0.9044	−0.014	0.8813	0.049	0.4174
2	*TRAF3IP1*	rs77008212	ADD	G	0.016	0.7425	−0.004	0.8595	0.017	0.1096	−0.034	0.2173	−0.031	0.0431*	0.014	0.1336
			DOM		0.014	0.7721	−0.004	0.8808	0.017	0.1151	−0.035	0.2064	−0.032	0.0400*	0.015	0.1362
			REC		0.381	0.5687	−0.138	0.6953	0.074	0.6190	0.107	0.7775	0.031	0.8844	0.039	0.7695
4	*PDGFRA*	rs17084051	ADD	A	0.000	0.9962	0.001	0.9536	0.004	0.4859	−0.027	0.0648	−0.008	0.3239	0.012	0.0197*
			DOM		−0.009	0.7732	0.005	0.7283	0.008	0.2173	−0.034	0.0440*	−0.007	0.4689	0.015	0.0110*
			REC		0.051	0.4791	−0.026	0.5024	−0.016	0.3257	−0.017	0.6750	−0.025	0.2841	0.007	0.6209
4	*PDGFRA*	rs2114039	ADD	C	−0.006	0.7967	0.005	0.6655	0.005	0.3034	−0.017	0.1715	−0.005	0.5030	0.011	0.0151*
			DOM		−0.016	0.5673	0.011	0.4548	0.009	0.1489	−0.026	0.1092	−0.006	0.5231	0.016	0.0050*
			REC		0.023	0.6624	−0.010	0.7283	−0.003	0.8275	−0.008	0.7820	−0.007	0.6880	0.006	0.5737
4	*PDGFRA*	rs2228230	ADD	T	0.008	0.7811	−0.005	0.7289	0.000	0.9552	−0.035	0.0382*	−0.026	0.0058*	0.008	0.2070
			DOM		0.012	0.7098	−0.006	0.7305	0.004	0.6311	−0.045	0.0170*	−0.034	0.0012**	0.009	0.1725
			REC		−0.021	0.8429	−0.008	0.8919	−0.031	0.1819	0.015	0.8081	0.017	0.6203	0.003	0.8889
4	*BMP3*	rs1353386	ADD	A	0.001	0.9611	−0.004	0.7859	−0.008	0.1764	−0.002	0.8929	−0.006	0.4806	−0.004	0.3981
			DOM		−0.008	0.7843	−0.002	0.8754	−0.012	0.0760	0.002	0.9236	−0.004	0.6599	−0.007	0.2168
			REC		0.060	0.4141	−0.016	0.6880	0.008	0.6464	−0.026	0.5310	−0.023	0.3364	0.009	0.5541
6	CASC15	rs4712652	ADD	G	−0.027	0.4100	0.014	0.4312	0.003	0.6734	0.005	0.7870	0.011	0.3024	−0.007	0.3092
			DOM		−0.020	0.5695	0.010	0.6018	0.005	0.4809	0.008	0.6840	0.015	0.1907	−0.007	0.2990
			REC		−0.210	0.1736	0.113	0.1631	−0.039	0.2569	−0.046	0.5992	−0.045	0.3544	−0.006	0.8475
6	CASC15	rs10946507	ADD	A	−0.004	0.8926	0.006	0.7007	0.004	0.5439	0.008	0.6314	0.015	0.1418	0.011	0.0764
			DOM		−0.007	0.8362	0.008	0.6689	0.005	0.4999	0.012	0.5357	0.013	0.2176	0.013	0.0530
			REC		0.027	0.8281	−0.002	0.9762	−0.001	0.9644	−0.025	0.7234	0.054	0.1760	−0.001	0.9802
6	*NHSL1*	rs4896367	ADD	C	−0.003	0.8940	0.004	0.7528	−0.002	0.7333	0.014	0.2585	0.018	0.0089*	−0.003	0.4780
			DOM		−0.017	0.5586	0.009	0.5416	−0.006	0.3778	0.011	0.5078	0.011	0.2275	−0.010	0.1013
			REC		0.033	0.4963	−0.008	0.7466	0.008	0.4727	0.038	0.1672	0.058	0.0002**	0.011	0.2436
6	*NHSL1*	rs4620141	ADD	C	0.015	0.4725	−0.011	0.3203	−0.011	0.0214*	−0.001	0.9341	−0.004	0.5617	−0.008	0.0645
			DOM		0.014	0.6637	−0.009	0.5737	−0.016	0.0190*	0.004	0.8273	−0.011	0.2692	−0.014	0.0240*
			REC		0.028	0.4444	−0.021	0.2696	−0.011	0.1904	−0.009	0.6847	0.003	0.7828	−0.005	0.5354
15	*FBN1*	rs9806595	ADD	C	0.007	0.7326	0.003	0.8200	0.002	0.7272	−0.010	0.4120	0.001	0.8939	0.005	0.2671
			DOM		0.013	0.6452	0.003	0.8573	0.003	0.6480	0.000	0.9966	0.004	0.6369	0.006	0.3218
			REC		−0.001	0.9859	0.005	0.8417	0.000	0.9964	−0.049	0.0730	−0.008	0.6211	0.008	0.4339
15	*FBN1*	rs25458	ADD	G	−0.003	0.9025	0.004	0.7372	−0.003	0.5931	0.000	0.9815	0.005	0.4366	0.005	0.2215
			DOM		0.003	0.9239	0.002	0.8908	0.000	0.9787	0.009	0.5937	0.008	0.4105	0.006	0.3291
			REC		−0.020	0.6703	0.013	0.6131	−0.013	0.2256	−0.025	0.3507	0.005	0.7367	0.010	0.2925

*CHR* = chromosome; *SNP* = single nucleotide polymorphism; *EA* = effect allele; *RA* = refractive astigmatism; *J0*_*(RA)*_ = J0 of refractive astigmatism; *J45*_*(RA)*_ = J45 of refractive astigmatism; *IA* = internal astigmatism; *J0*_*(IA)*_ = J0 of internal astigmatism; *J45*_*(IA)*_ = J45 of internal astigmatism; *ADD* = additive genetic model; *DOM* = dominant genetic model; *REC* = recessive genetic model

*P* values in bold with ** indicate study-wide significance level; * nominal significance level

The stratified analysis showed an association of *FMNL2* rs1579050 with J0_(RA)_ (additive: β = 0.132, *P* = 0.0014; dominant: β = 0.125, *P* = 0.0030; recessive: β = 0.953, *P* = 0.0107) in males, as well as *PDGFRA* rs2114039 with K2 (additive: β = 0.188, *P* = 0.0109; dominant: β = 0.282, *P* = 0.0034; recessive: β = 0.116, *P* = 0.4902), *PDGFRA* rs17084051 with IA (additive: β = −0.058, *P* = 0.0044; dominant: β = −0.079, *P* = 0.0014; recessive: β = −0.029, *P* = 0.6009), *PDGFRA* rs2228230 with IA (additive: β = −0.071, *P* = 0.0046; dominant: β = −0.083, *P* = 0.0023; recessive: β = −0.003, *P* = 0.9746) and J0_(IA)_ (additive: β = −0.049, *P* = 0.0004; dominant: β = −0.057, *P* = 0.0001; recessive: β = 0.001, *P* = 0.9855), and *FBN1* rs25458 with J45_(CA)_ (additive: β = −0.015, *P* = 0.0516; dominant: β = −0.007, *P* = 0.4769; recessive: β = −0.051, *P* = 0.0025) in females (Supplementary Figure 2). *PDGFRA* rs2228230 was also associated with IA (additive: β = −0.071, *P* = 0.0018; dominant: β = −0.083, *P* = 0.0011; recessive: β = −0.052, *P* = 0.5394) and J0_(IA)_ (additive: β = −0.039, *P* = 0.0026; dominant: β = −0.047, *P* = 0.0010; recessive: β = −0.009, *P* = 0.8580, Supplementary Figure 3) in 4–7-year-olds. No significant associations were observed for dichotomous astigmatism in the stratified analysis (Supplementary Figures 4 and 5).

There was a significant effect of the interaction between *FBN1* rs25458 and age on K1 (additive: *P* = 0.0350; dominant: *P* = 0.0031; recessive: *P* = 0.7661, Supplementary Table 4), and the effect of the interaction between *FBN1* rs9806595 and sex on J45_(CA)_ was also statistically significant (additive: *P* = 0.4130; dominant: *P* = 0.4553; recessive: *P* = 0.0026, Supplementary Table 4). Additionally, *FMNL2* rs1579050 and age interaction significantly affected J45_(IA)_ (additive: *P* = 0.0023; dominant: *P* = 0.0030; recessive: *P* = 0.1282, Supplementary Table 5).

## Discussion

Here, 14 candidate SNPs at/near nine loci previously identified to be associated with CR, CA, and RA in adults were evaluated for their associations with K1, K2, CR, CA, RA, IA, and vector components of astigmatism in Chinese children. *FMNL2* rs1579050 was associated with CA, J0_(CA)_, and dichotomous RA, whereas *NHSL1* rs4896367 showed associations with J0_(IA)_ and dichotomous IA. Moreover, *PDGFRA* rs2228230 was associated with J0_(IA)_. A dose–response relationship was found between the risk allele of *FMNL2* rs1579050 and susceptibility to CA and RA.

Aligning with the current findings, variants at/near *FMNL2* were associated with increasing severity of CA and RA in an adult GWAS, but at suggestive association levels [18]. The biological plausibility of *FMNL2*’s role in ocular structural disorders is further supported by its associations with primary open-angle glaucoma [37, 38] and related endophenotypes, including intraocular pressure (IOP) [39] and vertical cup-to-disc ratio [40]. This pleiotropy highlights *FMNL2* as a potential regulator of anterior segment architecture. The genetic associations are biologically plausible given the fundamental role of the *FMNL2* protein in actin cytoskeleton dynamics. The *FMNL2* gene encodes a formin-related protein, part of a highly conserved family of cytoskeletal remodelling proteins that play a role in a range of actin-dependent physiological processes [41]. Formins are characterised by the presence of formin homology 1 (FH1) and FH2 domains, which promote actin assembly [42]. The FH2 domain nucleates new actin filaments while remaining attached to their barbed ends, and it is preceded by the FH1 domain, which binds to profilin–actin complexes to recruit and deliver new actin subunits for filament elongation [42]. Actin microfilaments are a main component of the cytoskeleton in the human eye, distributed across various tissues, including those critical for refraction, such as the cornea and the lens [43, 44]. Genetic variations in *FMNL2* likely disrupt the cytoskeletal remodelling required for maintaining CR. This may occur through impaired actin assembly in corneal keratocytes, potentially leading to biomechanical instability and asymmetrical curvature—the hallmark of astigmatism. In the trabecular meshwork, the same mechanism of cytoskeletal dysregulation may impede aqueous humour outflow, increasing IOP—a major risk factor for glaucoma—and potentially influencing optic nerve head morphology. Cytoskeletal changes in the trabecular meshwork have been linked to corticosteroid-induced elevation of IOP [43].

We found significant associations of an *NHSL1* variant with J0_(IA)_ and an increased risk of dichotomous IA, and previous research has shown a suggestive association of the variant with a higher risk of CA in adult GWAS [19]. The associations of *NHSL1* variants with IA have not been investigated in adults, but the results suggest that *NHSL1* likely influences astigmatism risk through a mechanism that begins in early life and persists over time. *NHSL1* rs4896367 is a regulatory element situated within a predicted enhancer active across diverse cell types (Ensembl ID: ENSR6_863X8D), suggesting it modulates gene expression. However, its specific function and target gene in ocular tissues remain to be determined. The paralogous *NHS* gene provides a mechanistic candidate for the observed association in this study. *NHS* is a known regulator of actin cytoskeletal remodelling and is involved in lens development [45, 46]; mutations in *NHS* cause the Nance-Horan syndrome, which, among other features, manifests as bilateral cataract [47, 48], supporting the biological plausibility of *NHSL1*’s involvement in IA pathogenesis. *NHSL1* possibly influences IA through dysregulation of actin-mediated processes critical to lens morphogenesis or structural maintenance, potentially leading to refractive imperfections. Further functional investigation to elucidate the role of *NHSL1* variants in ocular development and astigmatism is warranted.

The correlation matrix revealed a pattern wherein increases in IA were accompanied by increases in CR and CA, while RA decreased, suggesting a compensatory interaction between IA and CA that may attenuate RA. This is consistent with prior reports of a compensatory increase in IA corresponding to increased CA [9, 49, 50]. Variants at/near *PDGFRA* were associated with K2, IA, and J0_(IA)_. Significant associations of *PDGFRA* SNPs with CR and CA have been reported in adults [17]. Given that most children in the cohort had WTR CA and ATR IA, the absence of any associations with RA may reflect the potential role of the *PDGFRA* gene in coordinating the scaling of J0_(IA)_ and CR to maintain a state of no astigmatism in children. This is supported by earlier work showing that ATR IA counteracts WTR CA in approximately 98% of children [50]. Previous studies have also demonstrated that *PDGFRA* is involved in the coordinated scaling of AL and CR, which potentially helps keep the eye in an emmetropic state during growth [51, 52]. The role of *PDGFRA* extends beyond astigmatism, with variants previously associated with a decreased risk of high myopia [53], as well as variations in lens thickness [54] and anterior chamber depth [54], highlighting its potential pleiotropic influence on ocular development. The *PDGFR-α*, encoded by the *PDGFRA* gene, is one of the isoforms of the platelet-derived growth factor (PDGF) receptors (PDGFRs), which are differentially expressed on the cell surfaces of various cell types [55, 56]. It engages several well-characterised signalling pathways, including Ras-MAPK, PI3K, and PLC-γ, which are known to be involved in multiple cellular and developmental responses [57]. The Ras-MAPK pathway is activated primarily through adaptor proteins Grb2 and Shc, with Grb2 binding to activated *PDGFR* and recruiting Sos1 [57]. Sos1 then activates Ras, initiating the MAPK cascade that promotes gene transcription and stimulates cell growth, differentiation, and migration [57]. Activation of the PI3K pathway by *PDGFR*s also enhances actin reorganization, directs cell movements, stimulates cell growth, and inhibits apoptosis, whereas the activation of PLC-γ by *PDGFR*s leads to increased cell growth and motility [57]. *PDGFR*s engage various other signalling molecules, including enzymes, adaptors, and transcription factors, which collectively promote cell growth, differentiation, and mitogenic responses through pathways involving Src TK, PKC-δ, JNK activation, and STAT phosphorylation [57]. In ocular tissues, *PDGF* receptor expression facilitates the proliferation and differentiation of lens epithelium into lens fibre cells, as well as mediates corneal fibroblast migration, matrix remodelling, and corneal wound healing [57–63]. Furthermore, treatment with *PDGF* was found to promote cell elongation in rabbit corneal keratocytes [64], and human corneal stromal cells exposed to *PDGF* similarly exhibited markedly elongated spindle shapes [65]. Taken together, *PDGFRA* may be a pleiotropic regulator of ocular biometry, affecting CR, lenticular dimensions, and overall refractive development, likely through shared pathways in cytoskeletal organization and growth signalling.

A principal advance of this study is the demonstration of significant genetic associations with the vector components of astigmatism. The genetic associations with J0 components, as seen for *FMNL2* with CA and RA, as well as *NHSL1/PDGFRA* with IA, suggest that the variants may influence biological processes that create or counteract a predominant toricity in the horizontal or vertical meridian. This could arise through asymmetrical eyelid pressure, cytoskeletal and extracellular matrix remodeling, or gradients in lenticular growth [41, 45, 46]. The role of the *FBN1* gene in extracellular matrix may also explain its association with J45_(CA)_. The *FBN1* gene encodes fibrillin-1 [66], a large glycoprotein that forms the core of microfibrils, providing structural support in connective tissues [67, 68]. It is highly expressed in the mouse cornea and lens capsule [69], and has been reported to be associated with a higher radius of curvature, reduced thickness, as well as disorganized and reduced density of elastic microfibril bundles of the cornea [70]. The *FBN1* gene has also been associated with higher deformation amplitude ratios and lower stress–strain index values, indicating reduced corneal stiffness [71]. We speculate that variants in *FBN1* might introduce torsional forces or asymmetries in the extracellular matrix of the cornea, leading to the misalignment captured by the J45 vector. This finding warrants confirmation and functional exploration to determine if *FBN1* variants have a direct effect on corneal matrix organization. The findings also imply that decomposing astigmatism into J0 and J45 moves genetic analysis beyond the question of how much astigmatism exists, allowing the exploration of what kind of astigmatism is present and providing a more precise mechanistic understanding of how the condition may be genetically regulated.

Our study revealed a genetic basis for childhood astigmatism, which highlights important clinical implications, particularly for early detection and monitoring strategies. The implications may extend beyond just refractive error to include the potential identification of children at risk for progressive corneal pathologies such as keratoconus. High and/or irregular astigmatism is frequently associated with keratoconus [72], and a higher prevalence of keratoconus has been reported in paediatric populations with significant refractive errors [73]. Besides, family history has been identified as an important risk factor for keratoconus, highlighting its heritable component [74]. First, our findings imply that the dose-dependent increase in astigmatism risk among children of astigmatic parents [8] may be driven not only by shared environment or lifestyle factors, but also by the passing of susceptibility alleles. Consequently, children with astigmatic parents should be considered a high-risk group warranting pre-emptive screening, even before exposure to significant influences by environmental factors. Additionally, the heritable components of corneal shape and internal optics we describe may act as quantitative endophenotypes, capturing the subclinical genetic liability that, in concert with environmental triggers like eye rubbing, could potentiate progressive ectasia in cases like keratoconus. Second, our analysis of vector components revealed that genes may have a greater impact on specific orientations of astigmatism rather than on its overall magnitude. Further exploration of this finding may help inform clinical decision-making, allowing clinicians and surgeons to adopt a more conservative treatment approach for astigmatic orientations that are more likely to shift due to non-genetic factors.

Not all the SNPs previously identified as having significant effects in adults were similarly significant in our cohort [17]. This aligns with prior studies on other types of refractive error, which have shown that the influence of SNPs can vary by age [75–78]. Specifically, some SNPs associated with early-onset effects exhibit stronger impacts later in life, while others display differing effects across various age groups [75–78]. For astigmatism, this age-specific effect seems to have a clear biological basis; the predominance of WTR astigmatism in children, primarily driven by eyelid tension, shifts toward ATR astigmatism in adults due to age-related lenticular and corneal changes [11, 79, 80]. By focusing on children, it is more likely that the observed astigmatism arose from a primary genetic origin, before its phenotypic expression was significantly influenced by environmental factors and age-related physiological changes.

Some associations of the variants at/near *FMNL2*, *PDGFRA*, and *FBN1* were modified by sex and/or age. Sex differences could plausibly arise from hormonal regulation, as sex hormone receptors are present in the cornea and lens [81–83]. Similarly, age-dependent effects may relate to developmental changes, such as those influenced by growth hormone [84].

The study has some strengths that enhance its contribution. First, it provides new insights into early genetic influences on astigmatism and supports the relevance of the significant SNPs across different developmental stages. Second, while the significant SNPs have been associated with CR, CA, and RA in adults, this study revealed their associations with IA and astigmatism vector components in children. This has not been previously reported. Lastly, the study provides evidence that nonadditive genetic influences on astigmatism occur in children. There are also limitations that may impact the interpretation of the results. First, the selection of SNPs was based on prior studies, which may lead to the omission of potentially significant and intriguing genetic variants. Moreover, while our findings suggest a developmental origin for astigmatism, we were not able to disentangle the effects of genetic predisposition from post-natal behavioural influences (e.g., eye rubbing) on the astigmatic measures. This remains a viable area for future investigations. Additional studies are also warranted on how genetic variants may modulate susceptibility to lifestyle or environmental risk factors for astigmatism.

## Conclusions

Genes previously reported to have associations with CR, CA and RA in adults showed overlapping associations with various types of astigmatism and their vector components in children. Overall, *FMNL2* rs1579050 was associated with CA and its J0 component, while *PDGFRA* rs2228230 and *NHSL1* rs4896367 were associated with J0_(IA)_ and/or IA. These results indicate that childhood astigmatism may have a heritable component and suggest that exploring alternative phenotypes related to astigmatism, such as vector components, along with employing nonadditive genetic models, could enhance the statistical power of genetic studies on astigmatism.

## Supplementary Information

Additional file 1

## Data Availability

The datasets used and/or analysed during the current study are available from the corresponding authors on reasonable request.

## Abbreviations

ATR: Against-the-rule
CA: Corneal astigmatism
CHS: Southern Chinese
COVID-19: Coronavirus disease 2019
CR: Corneal curvature
D: Diopter
FDR: False discovery rate
FH1: Formin homology 1
FH2: Formin homology 2
GWAS: Genome-wide association study
HWE: Hardy–Weinberg equilibrium
IA: Internal astigmatism
J0: Orthogonal/Cartesian astigmatism
J45: Oblique astigmatism
K1: Flat keratometry reading
K2: Steep keratometry reading
OR: Odds ratio
RA: Refractive astigmatism
SNP: Single-nucleotide polymorphism
WTR: With-the-rule
