# Supplementary material for: Gene polymorphisms associated with corneal curvature, astigmatism and its vector components in children
Source: Eye Vis (Lond). 2025 Dec 1;12:47. doi: 10.1186/s40662-025-00464-y (PMC12667156; doi:10.1186/s40662-025-00464-y)
Supplement: Supplementary file 1 — Additional file 1 [file 40662_2025_464_MOESM1_ESM.docx]

**Supplementary Material**

| **Supplementary Table 1.** Characteristics of selected SNPs | | | | | | | | | | |  |  |
| --- | --- | --- | --- | --- | --- | --- | --- | --- | --- | --- | --- | --- |
| **CHR** | **Position (GRch37)** | **Locus** | **SNP** | **Phenotype** | **EA** | **OA** | **Beta** | ***P* value** | **Transcript consequence (most severe)** | **Cohorts** | **Age (years)** | **Reference** |
| 1 | 213817311 | *RPS6KC1* | rs12144639 | CA | A | G | 0.530 | 4.75×10^−6^ | Intron variant | European, Chinese | 6.09 ± 0.4 to 73.3 ± 7.6 | Shah et al., 2018 [1] |
| 1 | 219788519 | *ZC3H11B* | rs7525202 |  |  |  |  |  | Intergenic variant |  |  |  |
| 2 | 153364527 | *FMNL2* | rs1579050 | CA | A | G | 0.015 | 3.00×10^−7^ | Intron variant | European (UK) | 58.2 ± 7.90 | Shah and Guggenheim, 2018 [2] |
| 2 | 239307113 | *TRAF3IP1* | rs77008212 | RA | A | G | –0.026 | 4.70×10^−7^ | Intron variant | European (UK) | 58.2 ± 7.90 | Shah and Guggenheim, 2018 [2] |
| 4 | 55087581 | *PDGFRA* | rs17084051 | CR | A | - | –0.126 | 4.49×10^−14^ | Non-coding transcript exon variant | Singaporean (Chinese, Malay, Indian) | 41.6 ± 20.82 | Guggenheim et al., 2013 [3] |
|  |  |  |  | CR | A | - | –0.153 | 1.00×10^−5^ |  | Malay Singaporean | 58 ± 10.82 | Han et al., 2011 [4] |
| 4 | 55092626 | *PDGFRA* | rs2114039 | CR | C | - | 0.107 | 1.48×10^−11^ | Intron variant | Singaporean (Chinese, Malay, Indian) | 41.6 ± 20.82 | Guggenheim et al., 2013 [3] |
| 4 | 55152040 | *PDGFRA* | rs2228230 | CA | T | - | 1.240 | 1.43×10^−6^ | Synonymous variant | Singaporean (Chinese, Malay, Indian) | 7.50 ± 3.80 to 57.70 ± 10.70 | Fan et al., 2011 [5] |
| 4 | 81947080 | *BMP3* | rs1353386 | CA | A | C | 0.019 | 1.00×10^−6^ | Intergenic variant | European (UK) | 58.2 ± 7.90 | Shah and Guggenheim, 2018 [2] |
| 6 | 22078615 | CASC15 | rs4712652 | RA | A | G | 1.097 | 3.13×10^−6^ | Intron variant | European | 56.55 ± 13.36 | Li et al., 2015 [6] |
| 6 | 22100367 | CASC15 | rs10946507 | RA | A | G | 0.930 | 2.15×10^−9^ | Intron variant | European (UK) | 58.2 ± 7.90 | Shah and Guggenheim, 2018 [2] |
| 6 | 138807281 | *NHSL1* | rs4896367 | CA | T | C | 1.090 | 9.75×10^−6^ | Intron variant | Asian, European | 51.37 ± 22.43 | Shah et al., 2018 [1] |
| 6 | 138869568 | *NHSL1* | rs4620141 | CR | T | C | –0.015 | 3.35×10^−6^ | Intron variant | Asian | 47.67 ± 17.8 | Fan et al., 2020 [7] |
|  |  |  |  | CR | T | C | –0.014 | 1.12×10^−11^ |  | Asian, European | 49.10 ± 21.1 | Fan et al., 2020 [7] |
|  |  |  |  | CR | T | C | –0.013 | 2.90×10^−7^ |  | European | 49.80 ± 22.5 | Fan et al., 2020 [7] |
| 15 | 48755168 | *FBN1* | rs9806595 | CR | T | C | –0.018 | 1.49×10^−7^ | Intron variant | Asian | 47.67 ± 17.8 | Fan et al., 2020 [7] |
|  |  |  |  | CR | T | C | –0.012 | 2.23×10^−8^ |  | Asian, European | 49.10 ± 21.1 | Fan et al., 20209 [7] |
| 15 | 48797307 | *FBN1* | rs25458 |  |  |  |  |  | Missense variant |  |  |  |
|  | | | | | | | | | | |  |  |

CHR = chromosome; SNP = single nucleotide polymorphism; EA = effect allele; OA = other allele; CR = corneal radius of curvature; CA = corneal astigmatism; RA = refractive astigmatism

We selected loci/genes with overlapping associations with CR, CA, and RA. While the specific SNPs listed in Supplemental Table 1 are individually associated with only one phenotype, each of their respective loci harbours other SNPs associated with the remaining clinical parameters. These other SNPs were not included as they did not meet our selection criteria. Furthermore, in Supplemental Table 1, if a phenotype and other details are not listed for a given SNP, it indicates that the SNP we selected is not the one originally reported for the missing phenotype. Instead, the selected SNP is a proxy that is in linkage disequilibrium with the original, reported variant. These other SNPs were not selected as they did not meet our specific criteria.

| **Supplementary Table 2**. Hardy-Weinberg Equilibrium test results for all genotyped SNPs | | | | | | | | |
| --- | --- | --- | --- | --- | --- | --- | --- | --- |
| **CHR** | **SNP** | **TEST** | **A1** | **A2** | **GENO** | **O(HET)** | **E(HET)** | ***P*** |
| 1 | rs12144639 | ALL(QT) | A | G | 101/725/1341 | 0.335 | 0.336 | 0.7985 |
| 1 | rs7525202 | ALL(QT) | G | A | 157/860/1150 | 0.397 | 0.395 | 0.8704 |
| 2 | rs1579050 | ALL(QT) | G | A | 5/153/2009 | 0.071 | 0.072 | 0.2284 |
| 2 | rs77008212 | ALL(QT) | G | A | 1/210/1956 | 0.097 | 0.093 | 0.0601 |
| 4 | rs17084051 | ALL(QT) | A | C | 89/679/1399 | 0.313 | 0.317 | 0.5430 |
| 4 | rs2114039 | ALL(QT) | C | T | 179/852/1136 | 0.393 | 0.403 | 0.2858 |
| 4 | rs2228230 | ALL(QT) | T | C | 41/511/1615 | 0.236 | 0.236 | 0.9275 |
| 4 | rs1353386 | ALL(QT) | A | C | 85/690/1392 | 0.318 | 0.318 | 1.0000 |
| 6 | rs4712652 | ALL(QT) | G | A | 19/452/1696 | 0.209 | 0.201 | 0.0681 |
| 6 | rs10946507 | ALL(QT) | A | G | 30/491/1646 | 0.227 | 0.222 | 0.3834 |
| 6 | rs4896367 | ALL(QT) | C | T | 217/910/1040 | 0.420 | 0.428 | 0.3934 |
| 6 | rs4620141 | ALL(QT) | C | T | 407/1083/677 | 0.500 | 0.492 | 0.4853 |
| 15 | rs9806595 | ALL(QT) | C | T | 216/940/1011 | 0.434 | 0.433 | 0.9604 |
| 15 | rs25458 | ALL(QT) | G | A | 218/949/1000 | 0.438 | 0.435 | 0.7671 |
| CHR = chromosome; SNP = single nucleotide polymorphism; A1 = minor allele; A2 = major allele; GENO = genotype counts (A1A1/A1A2/A2A2); O(HET) = observed heterozygote frequency; E(HET) = expected heterozygote frequency; QT = quantitative phenotype | | | | | | | | |

| **Supplemental Table 3.** Results of Cochran-Armitage Trend Test | | | | | | | | | | | | | | | | |
| --- | --- | --- | --- | --- | --- | --- | --- | --- | --- | --- | --- | --- | --- | --- | --- | --- |
|  |  |  |  |  | **Corneal Astigmatism** | | | | **Refractive Astigmatism** | | | | **Internal Astigmatism** | | | |
| **CHR** | **Locus** | **SNP** | **EA** | **OA** | **AFF** | **UNAFF** | **χ^2^** | ***P*** | **AFF** | **UNAFF** | **χ^2^** | ***P*** | **AFF** | **UNAFF** | **χ^2^** | ***P*** |
| 1 | *RPS6KC1* | rs12144639 | A | G | 716/2584 | 211/821 | 0.728 | 0.3935 | 321/1229 | 606/2164 | 0.801 | 0.3709 | 355/1263 | 558/2068 | 0.283 | 0.5947 |
| 1 | *ZC3H11B* | rs7525202 | G | A | 885/2415 | 288/744 | 0.474 | 0.4912 | 436/1114 | 734/2036 | 1.343 | 0.2465 | 456/1162 | 700/1926 | 1.181 | 0.2771 |
| 2 | *FMNL2* | rs1579050 | G | A | 140/3160 | 23/1009 | 8.592 | **0.0034**** | 77/1473 | 86/2684 | 9.274 | **0.0023**** | 69/1549 | 90/2536 | 1.896 | 0.1685 |
| 2 | *TRAF3IP1* | rs77008212 | G | A | 167/3133 | 45/987 | 0.864 | 0.3527 | 79/1471 | 133/2637 | 0.194 | 0.6597 | 93/1525 | 117/2509 | 3.711 | 0.0540 |
| 4 | *PDGFRA* | rs17084051 | A | C | 662/2638 | 194/838 | 0.780 | 0.3772 | 311/1239 | 546/2224 | 0.077 | 0.7812 | 340/1278 | 500/2126 | 2.428 | 0.1192 |
| 4 | *PDGFRA* | rs2114039 | C | T | 938/2362 | 271/761 | 1.788 | 0.1811 | 429/1121 | 779/1991 | 0.096 | 0.7571 | 474/1144 | 712/1914 | 2.322 | 0.1276 |
| 4 | *PDGFRA* | rs2228230 | T | C | 456/2844 | 137/895 | 0.196 | 0.6581 | 207/1343 | 385/2385 | 0.248 | 0.6182 | 236/1382 | 345/2281 | 1.775 | 0.1828 |
| 4 | *BMP3* | rs1353386 | A | C | 664/2636 | 195/837 | 0.744 | 0.3885 | 307/1243 | 551/2219 | 0.005 | 0.9463 | 313/1305 | 533/2093 | 0.569 | 0.4506 |
| 6 | CASC15 | rs4712652 | G | A | 380/2920 | 110/922 | 0.599 | 0.4391 | 181/1369 | 309/2461 | 0.281 | 0.5961 | 187/1431 | 300/2326 | 0.018 | 0.8925 |
| 6 | CASC15 | rs10946507 | A | G | 427/2873 | 124/908 | 0.617 | 0.4320 | 193/1357 | 355/2415 | 0.122 | 0.7274 | 209/1409 | 328/2298 | 0.169 | 0.6814 |
| 6 | *NHSL1* | rs4896367 | C | T | 998/2302 | 344/688 | 3.452 | 0.0632 | 490/1060 | 851/1919 | 0.362 | 0.5472 | 465/1153 | 853/1773 | 6.449 | 0.0110* |
| 6 | *NHSL1* | rs4620141 | C | T | 1441/1859 | 455/577 | 0.058 | 0.8099 | 695/855 | 1197/1573 | 1.082 | 0.2983 | 710/908 | 1147/1479 | 0.017 | 0.8965 |
| 15 | *FBN1* | rs9806595 | C | T | 1047/2253 | 323/709 | 0.067 | 0.7956 | 466/1084 | 900/1870 | 2.712 | 0.0996 | 510/1108 | 834/1792 | 0.027 | 0.8706 |
| 15 | *FBN1* | rs25458 | G | A | 1055/2245 | 328/704 | 0.013 | 0.9102 | 476/1074 | 902/1868 | 1.581 | 0.2086 | 503/1115 | 855/1771 | 1.003 | 0.3166 |
| CHR = chromosome; SNP = single nucleotide polymorphism; EA = effect allele; OA = other allele; AFF = affected; UNAFF = unaffected; χ^2^ = Chi-squared  *P* values in bold with ** indicate study**-**wide significance level; ^*^ nominal significance level | | | | | | | | | | | | | | | | |

| Supplementary Table 4. *P* values for the effects of SNP interactions with age and sex on corneal curvature and corneal astigmatism. | | | | | | | | | | | | | | | | |
| --- | --- | --- | --- | --- | --- | --- | --- | --- | --- | --- | --- | --- | --- | --- | --- | --- |
|  |  |  |  |  | **K1** | | **K2** | | **CR** | | **CA** | | **J0_(CA)_** | | **J45_(CA)_** | |
| CHR | **LOCUS** | **SNP** | **Model** | **EA** | **Age** | **Sex** | **Age** | **Sex** | **Age** | **Sex** | **Age** | **Sex** | **Age** | **Sex** | **Age** | **Sex** |
| 1 | *RPS6KC1* | rs12144639 | ADD | A | 0.4054 | 0.5864 | 0.9863 | 0.6662 | 0.6958 | 0.6185 | 0.2119 | 0.7993 | 0.1736 | 0.9659 | 0.3963 | 0.8761 |
|  |  |  | DOM |  | 0.4137 | 0.4897 | 0.9043 | 0.5553 | 0.7432 | 0.5123 | 0.0880 | 0.9452 | 0.0680 | 0.9280 | 0.5035 | 0.3732 |
|  |  |  | REC |  | 0.6802 | 0.9288 | 0.8203 | 0.8738 | 0.7469 | 0.8966 | 0.6283 | 0.5817 | 0.6495 | 0.9203 | 0.4230 | 0.1043 |
| 1 | *ZC3H11B* | rs7525202 | ADD | G | 0.1876 | 0.6999 | 0.1470 | 0.9961 | 0.1532 | 0.8549 | 0.3522 | 0.4362 | 0.1217 | 0.6644 | 0.2454 | 0.2307 |
|  |  |  | DOM |  | 0.4442 | 0.9869 | 0.3585 | 0.7380 | 0.3838 | 0.8485 | 0.3830 | 0.4693 | 0.2507 | 0.4430 | 0.0770 | 0.1569 |
|  |  |  | REC |  | 0.0810 | 0.3744 | 0.0760 | 0.5845 | 0.0700 | 0.4674 | 0.5512 | 0.5908 | 0.1203 | 0.7365 | 0.5686 | 0.8308 |
| 2 | *FMNL2* | rs1579050 | ADD | G | 0.6521 | 0.1234 | 0.7125 | 0.0780 | 0.6755 | 0.0880 | 0.9977 | 0.3741 | 0.9609 | 0.4981 | 0.0890 | 0.5975 |
|  |  |  | DOM |  | 0.4445 | 0.1729 | 0.4291 | 0.1556 | 0.4233 | 0.1518 | 0.7481 | 0.6294 | 0.7998 | 0.7808 | 0.1301 | 0.4302 |
|  |  |  | REC |  | 0.3097 | 0.4676 | 0.1161 | 0.0660 | 0.1773 | 0.1755 | 0.1320 | 0.0075^*^ | 0.1603 | 0.0110^*^ | 0.0870 | 0.0530 |
| 2 | *TRAF3IP1* | rs77008212 | ADD | G | 0.0210^*^ | 0.6643 | 0.0500 | 0.5916 | 0.0300^*^ | 0.6153 | 0.9296 | 0.9832 | 0.9802 | 0.8070 | 0.7790 | 0.4763 |
|  |  |  | DOM |  | 0.0240^*^ | 0.6985 | 0.0580 | 0.6373 | 0.0340^*^ | 0.6567 | 0.9858 | 0.9355 | 0.9668 | 0.7624 | 0.7888 | 0.4647 |
|  |  |  | REC |  | NA | NA | NA | NA | NA | NA | NA | NA | NA | NA | NA | NA |
| 4 | *PDGFRA* | rs17084051 | ADD | A | 0.5340 | 0.1738 | 0.6279 | 0.1173 | 0.5723 | 0.1306 | 0.7259 | 0.4183 | 0.5619 | 0.4601 | 0.2018 | 0.5017 |
|  |  |  | DOM |  | 0.7270 | 0.2444 | 0.9967 | 0.1697 | 0.8647 | 0.1895 | 0.3613 | 0.4539 | 0.2016 | 0.5663 | 0.1643 | 0.7138 |
|  |  |  | REC |  | 0.3467 | 0.2633 | 0.1657 | 0.2145 | 0.2256 | 0.2233 | 0.2264 | 0.5564 | 0.1459 | 0.4208 | 0.7727 | 0.2942 |
| 4 | *PDGFRA* | rs2114039 | ADD | C | 0.3143 | 0.2094 | 0.4730 | 0.1215 | 0.3804 | 0.1465 | 0.6982 | 0.4346 | 0.7255 | 0.6032 | 0.4511 | 0.8726 |
|  |  |  | DOM |  | 0.3629 | 0.1653 | 0.3856 | 0.0820 | 0.3613 | 0.1053 | 0.8694 | 0.3819 | 0.9218 | 0.6323 | 0.8727 | 0.5759 |
|  |  |  | REC |  | 0.5184 | 0.6157 | 0.9720 | 0.5643 | 0.7414 | 0.5774 | 0.2114 | 0.7505 | 0.3085 | 0.6618 | 0.1408 | 0.5338 |
| 4 | *PDGFRA* | rs2228230 | ADD | T | 0.3177 | 0.6795 | 0.6039 | 0.8061 | 0.4443 | 0.7390 | 0.3385 | 0.8803 | 0.6022 | 0.8785 | 0.2444 | 0.4240 |
|  |  |  | DOM |  | 0.3725 | 0.7712 | 0.7016 | 0.8691 | 0.5228 | 0.8179 | 0.2920 | 0.9149 | 0.5001 | 0.8820 | 0.1786 | 0.2954 |
|  |  |  | REC |  | 0.4174 | 0.9207 | 0.4651 | 0.7110 | 0.4295 | 0.8020 | 0.9913 | 0.5329 | 0.6874 | 0.7037 | 0.8450 | 0.7030 |
| 4 | *BMP3* | rs1353386 | ADD | A | 0.8316 | 0.1203 | 0.8877 | 0.1321 | 0.9800 | 0.1159 | 0.4914 | 0.5413 | 0.8201 | 0.7051 | 0.7824 | 0.6221 |
|  |  |  | DOM |  | 0.7634 | 0.0598 | 0.8425 | 0.0730 | 0.9712 | 0.0590 | 0.3338 | 0.5094 | 0.5831 | 0.6562 | 0.6629 | 0.5437 |
|  |  |  | REC |  | 0.8768 | 0.6865 | 0.9570 | 0.7869 | 0.9640 | 0.7322 | 0.6996 | 0.8592 | 0.4910 | 0.9863 | 0.7952 | 0.9837 |
| 6 | CASC15 | rs4712652 | ADD | G | 0.5537 | 0.6948 | 0.9376 | 0.9370 | 0.7430 | 0.8165 | 0.3738 | 0.5361 | 0.8953 | 0.8633 | 0.9836 | 0.5650 |
|  |  |  | DOM |  | 0.4920 | 0.4479 | 0.7635 | 0.6930 | 0.6203 | 0.5610 | 0.5646 | 0.5261 | 0.8555 | 0.8999 | 0.6629 | 0.5680 |
|  |  |  | REC |  | 0.7917 | 0.1288 | 0.3258 | 0.1665 | 0.5062 | 0.1367 | 0.0970 | 0.9105 | 0.1463 | 0.7959 | 0.0590 | 0.8651 |
| 6 | CASC15 | rs10946507 | ADD | A | 0.8866 | 0.0320^*^ | 0.7643 | 0.0130^*^ | 0.8160 | 0.0170^*^ | 0.9902 | 0.1898 | 0.9750 | 0.2194 | 0.7988 | 0.1131 |
|  |  |  | DOM |  | 0.6284 | 0.0910 | 0.5683 | 0.0550 | 0.5856 | 0.0630 | 0.9284 | 0.3433 | 0.8682 | 0.3977 | 0.5948 | 0.1579 |
|  |  |  | REC |  | 0.2696 | 0.0190^*^ | 0.4292 | 0.0047^*^ | 0.3350 | 0.0075^*^ | 0.6686 | 0.0820 | 0.6212 | 0.0710 | 0.3619 | 0.2597 |
| 6 | *NHSL1* | rs4896367 | ADD | C | 0.6932 | 0.3810 | 0.6364 | 0.3080 | 0.6537 | 0.3273 | 0.7959 | 0.9881 | 0.6925 | 0.5195 | 0.2701 | 0.1261 |
|  |  |  | DOM |  | 0.3983 | 0.5366 | 0.3644 | 0.5368 | 0.3665 | 0.5251 | 0.8940 | 0.8554 | 0.7686 | 0.6836 | 0.3913 | 0.2683 |
|  |  |  | REC |  | 0.5756 | 0.3653 | 0.6436 | 0.2330 | 0.6012 | 0.2764 | 0.7663 | 0.7725 | 0.6638 | 0.4228 | 0.3112 | 0.1264 |
| 6 | *NHSL1* | rs4620141 | ADD | C | 0.7707 | 0.8108 | 0.2576 | 0.8632 | 0.4474 | 0.9833 | 0.1217 | 0.4392 | 0.2559 | 0.3612 | 0.6967 | 0.1089 |
|  |  |  | DOM |  | 0.3346 | 0.9477 | 0.6495 | 0.7200 | 0.8292 | 0.8198 | 0.0054^*^ | 0.5314 | 0.0270^*^ | 0.4338 | 0.5513 | 0.0220^*^ |
|  |  |  | REC |  | 0.0980 | 0.6100 | 0.1402 | 0.9139 | 0.1089 | 0.7606 | 0.5975 | 0.5060 | 0.5610 | 0.4586 | 0.9919 | 0.8673 |
| 15 | *FBN1* | rs9806595 | ADD | C | 0.0450^*^ | 0.1352 | 0.1968 | 0.1864 | 0.0950 | 0.1492 | 0.4551 | 0.7079 | 0.2623 | 0.1506 | 0.7608 | 0.4130 |
|  |  |  | DOM |  | 0.0660 | 0.0400^*^ | 0.3474 | 0.0280^*^ | 0.1622 | 0.0280^*^ | 0.1857 | 0.3328 | 0.1927 | 0.0790 | 0.7086 | 0.4553 |
|  |  |  | REC |  | 0.1580 | 0.9521 | 0.1804 | 0.4923 | 0.1577 | 0.6856 | 0.5449 | 0.4809 | 0.7874 | 0.7820 | 0.9560 | **0.0026^**^** |
| 15 | *FBN1* | rs25458 | ADD | G | 0.0350^*^ | 0.1974 | 0.0660 | 0.1954 | 0.0430^*^ | 0.1838 | 0.7973 | 0.4943 | 0.7964 | 0.0870 | 0.5523 | 0.1222 |
|  |  |  | DOM |  | **0.0031^**^** | 0.5349 | 0.0087^*^ | 0.4717 | 0.0043^*^ | 0.4885 | 0.7937 | 0.6419 | 0.8322 | 0.3072 | 0.3511 | 0.6289 |
|  |  |  | REC |  | 0.7661 | 0.0780 | 0.7343 | 0.1053 | 0.7422 | 0.0830 | 0.8564 | 0.4424 | 0.3753 | 0.0390^*^ | 0.8466 | 0.0120^*^ |
| CHR = chromosome; SNP = single nucleotide polymorphism; EA = effect allele; K1 = flat keratometry reading; K2 = steep keratometry reading; CR = mean keratometry reading; CA = corneal astigmatism; J0_(CA)_ = J0 of corneal astigmatism; J45_(CA)_ = J45 of corneal astigmatism; ADD = additive genetic model; DOM = dominant genetic model; REC = recessive genetic model  *P* values in bold with ** indicate study-wide significance level; ^*^ nominal significance level | | | | | | | | | | | | | | | | |

| Supplementary Table 5. *P* values for the effects of SNP interactions with age and sex on refractive and internal astigmatism | | | | | | | | | | | | | | | | |
| --- | --- | --- | --- | --- | --- | --- | --- | --- | --- | --- | --- | --- | --- | --- | --- | --- |
|  |  |  |  |  | **RA** | | **J0_(RA)_** | | **J45_(RA)_** | | **IA** | | **J0_(IA)_** | | **J45_(IA)_** | |
| CHR | **LOCUS** | **SNP** | **Model** | **EA** | **Age** | **Sex** | **Age** | **Sex** | **Age** | **Sex** | **Age** | **Sex** | **Age** | **Sex** | **Age** | **Sex** |
| 1 | *RPS6KC1* | rs12144639 | ADD | A | 0.1532 | 0.9861 | 0.2502 | 0.8788 | 0.7656 | 0.7393 | 0.5303 | 0.5009 | 0.6884 | 0.4894 | 0.5209 | 0.4942 |
|  |  |  | DOM |  | 0.0730 | 0.8718 | 0.1050 | 0.7172 | 0.8387 | 0.9447 | 0.4766 | 0.5355 | 0.7246 | 0.4550 | 0.5583 | 0.2978 |
|  |  |  | REC |  | 0.8329 | 0.7529 | 0.5689 | 0.6768 | 0.7124 | 0.2743 | 0.9286 | 0.6679 | 0.7553 | 0.8431 | 0.6872 | 0.6171 |
| 1 | *ZC3H11B* | rs7525202 | ADD | G | 0.5055 | 0.1933 | 0.5436 | 0.2549 | 0.4068 | 0.3349 | 0.4822 | 0.7431 | 0.1823 | 0.1169 | 0.0330^*^ | 0.7954 |
|  |  |  | DOM |  | 0.3262 | 0.1925 | 0.4672 | 0.1853 | 0.7153 | 0.3965 | 0.3706 | 0.8543 | 0.5473 | 0.2034 | 0.0190^*^ | 0.4953 |
|  |  |  | REC |  | 0.7894 | 0.4663 | 0.9391 | 0.7747 | 0.1959 | 0.4711 | 0.9948 | 0.2834 | 0.0380^*^ | 0.1767 | 0.5177 | 0.5087 |
| 2 | *FMNL2* | rs1579050 | ADD | G | 0.1400 | 0.0461 | 0.3096 | 0.0420^*^ | 0.2968 | 0.4479 | 0.3019 | 0.0900 | 0.0940 | 0.0180^*^ | **0.0023^**^** | 0.7343 |
|  |  |  | DOM |  | 0.0550 | 0.0980 | 0.1571 | 0.0862 | 0.2432 | 0.4168 | 0.2621 | 0.0630 | 0.0630 | 0.0140^*^ | **0.0030^**^** | 0.9182 |
|  |  |  | REC |  | 0.0860 | 0.0080^*^ | 0.1064 | 0.0120^*^ | 0.6559 | 0.7712 | 0.8111 | 0.7238 | 0.6752 | 0.9372 | 0.1282 | 0.0490^*^ |
| 2 | *TRAF3IP1* | rs77008212 | ADD | G | 0.9955 | 0.7013 | 0.9197 | 0.6392 | 0.9235 | 0.1034 | 0.3273 | 0.7303 | 0.7798 | 0.6679 | 0.7027 | 0.3198 |
|  |  |  | DOM |  | 0.9688 | 0.6712 | 0.9446 | 0.6176 | 0.9110 | 0.0980 | 0.2994 | 0.7588 | 0.7395 | 0.6964 | 0.7010 | 0.3164 |
|  |  |  | REC |  | NA | NA | NA | NA | NA | NA | NA | NA | NA | NA | NA | NA |
| 4 | *PDGFRA* | rs17084051 | ADD | A | 0.9172 | 0.8971 | 0.8737 | 0.7128 | 0.3749 | 0.4323 | 0.5106 | 0.0374^*^ | 0.1822 | 0.1589 | 0.7053 | 0.7416 |
|  |  |  | DOM |  | 0.7447 | 0.7555 | 0.7697 | 0.4912 | 0.2091 | 0.6565 | 0.4565 | 0.0170^*^ | 0.1037 | 0.0910 | 0.7855 | 0.7867 |
|  |  |  | REC |  | 0.6196 | 0.6588 | 0.2427 | 0.5045 | 0.6093 | 0.1909 | 0.9648 | 0.7650 | 0.9136 | 0.9446 | 0.6354 | 0.8858 |
| 4 | *PDGFRA* | rs2114039 | ADD | C | 0.9347 | 0.9460 | 0.9444 | 0.5390 | 0.7644 | 0.4121 | 0.6316 | 0.0290^*^ | 0.4111 | 0.0800 | 0.5676 | 0.3514 |
|  |  |  | DOM |  | 0.4333 | 0.6888 | 0.5385 | 0.7240 | 0.8126 | 0.8024 | 0.8119 | 0.0320^*^ | 0.2434 | 0.1632 | 0.9551 | 0.4926 |
|  |  |  | REC |  | 0.1006 | 0.6065 | 0.1896 | 0.4623 | 0.7448 | 0.1222 | 0.4825 | 0.1953 | 0.8399 | 0.1137 | 0.1596 | 0.2707 |
| 4 | *PDGFRA* | rs2228230 | ADD | T | 0.5785 | 0.1919 | 0.9960 | 0.1646 | 0.3057 | 0.5772 | 0.0520 | 0.0620 | 0.2413 | 0.0370^*^ | 0.8537 | 0.9860 |
|  |  |  | DOM |  | 0.5920 | 0.2772 | 0.8301 | 0.1749 | 0.3334 | 0.3192 | 0.0650 | 0.0648 | 0.2868 | 0.0460^*^ | 0.5732 | 0.8481 |
|  |  |  | REC |  | 0.7739 | 0.2644 | 0.5055 | 0.5500 | 0.6165 | 0.3926 | 0.3629 | 0.8344 | 0.5456 | 0.7576 | 0.2703 | 0.7248 |
| 4 | *BMP3* | rs1353386 | ADD | A | 0.5115 | 0.8680 | 0.4280 | 0.9637 | 0.7293 | 0.4743 | 0.7899 | 0.1988 | 0.3835 | 0.4435 | 0.6301 | 0.0987 |
|  |  |  | DOM |  | 0.5855 | 0.9797 | 0.3803 | 0.9847 | 0.7054 | 0.4854 | 0.6236 | 0.1870 | 0.4984 | 0.3974 | 0.3767 | 0.1102 |
|  |  |  | REC |  | 0.5673 | 0.7996 | 0.8906 | 0.8858 | 0.9772 | 0.9876 | 0.6649 | 0.5406 | 0.3961 | 0.8171 | 0.4276 | 0.5659 |
| 6 | CASC15 | rs4712652 | ADD | G | 0.1907 | 0.7311 | 0.3910 | 0.9442 | 0.4279 | 0.8392 | 0.5735 | 0.1416 | 0.2338 | 0.9213 | 0.4478 | 0.6750 |
|  |  |  | DOM |  | 0.2414 | 0.7902 | 0.4379 | 0.8878 | 0.2400 | 0.8293 | 0.7707 | 0.1886 | 0.1197 | 0.9236 | 0.4954 | 0.6906 |
|  |  |  | REC |  | 0.3225 | 0.6606 | 0.5353 | 0.7696 | 0.1373 | 0.9932 | 0.1721 | 0.2710 | 0.1951 | 0.3709 | 0.5739 | 0.8369 |
| 6 | CASC15 | rs10946507 | ADD | A | 0.5331 | 0.4635 | 0.5187 | 0.7907 | 0.6717 | 0.1312 | 0.9800 | 0.3991 | 0.4133 | 0.1156 | 0.4592 | 0.8986 |
|  |  |  | DOM |  | 0.5887 | 0.6569 | 0.6348 | 0.9378 | 0.9339 | 0.1550 | 0.9315 | 0.3866 | 0.7024 | 0.1761 | 0.4638 | 0.9386 |
|  |  |  | REC |  | 0.6312 | 0.1977 | 0.4191 | 0.4460 | 0.1547 | 0.4011 | 0.8040 | 0.8035 | 0.0590 | 0.1931 | 0.7371 | 0.8942 |
| 6 | *NHSL1* | rs4896367 | ADD | C | 0.8592 | 0.3766 | 0.5163 | 0.2459 | 0.4829 | 0.4500 | 0.0190^*^ | 0.1248 | 0.4085 | 0.3145 | 0.5522 | 0.2714 |
|  |  |  | DOM |  | 0.5017 | 0.3057 | 0.3457 | 0.2549 | 0.4461 | 0.6871 | 0.0320^*^ | 0.2270 | 0.1541 | 0.2315 | 0.7745 | 0.3101 |
|  |  |  | REC |  | 0.4893 | 0.7915 | 0.9091 | 0.5016 | 0.8170 | 0.3347 | 0.1223 | 0.1823 | 0.5365 | 0.8985 | 0.3699 | 0.4439 |
| 6 | *NHSL1* | rs4620141 | ADD | C | 0.3933 | 0.1428 | 0.4809 | 0.1028 | 0.7886 | 0.1193 | 0.1603 | 0.0623 | 0.2982 | 0.2159 | 0.4494 | 0.9303 |
|  |  |  | DOM |  | 0.0590 | 0.0560 | 0.0480^*^ | 0.0410^*^ | 0.9386 | 0.0530 | 0.1596 | 0.0270^*^ | 0.6188 | 0.0680 | 0.5820 | 0.7465 |
|  |  |  | REC |  | 0.4782 | 0.7193 | 0.2778 | 0.6124 | 0.5521 | 0.6262 | 0.4067 | 0.4731 | 0.2009 | 0.9859 | 0.4734 | 0.6040 |
| 15 | *FBN1* | rs9806595 | ADD | C | 0.7894 | 0.7147 | 0.8403 | 0.9855 | 0.3432 | 0.3091 | 0.5800 | 0.8347 | 0.0410^*^ | 0.0870 | 0.1799 | 0.7318 |
|  |  |  | DOM |  | 0.9276 | 0.9215 | 0.9525 | 0.5353 | 0.6309 | 0.5674 | 0.1568 | 0.9882 | 0.0300^*^ | 0.2028 | 0.3825 | 0.0780 |
|  |  |  | REC |  | 0.6452 | 0.5426 | 0.5912 | 0.2858 | 0.2098 | 0.2105 | 0.2293 | 0.7135 | 0.4160 | 0.0887 | 0.1434 | 0.0280^*^ |
| 15 | *FBN1* | rs25458 | ADD | G | 0.8347 | 0.6236 | 0.8635 | 0.1999 | 0.7784 | 0.6005 | 0.2063 | 0.0890 | 0.9521 | 0.8041 | 0.6363 | 0.2245 |
|  |  |  | DOM |  | 0.6514 | 0.8569 | 0.7684 | 0.3923 | 0.3159 | 0.7534 | 0.1888 | 0.2215 | 0.4818 | 0.9559 | 0.9145 | 0.8732 |
|  |  |  | REC |  | 0.7545 | 0.4322 | 0.8980 | 0.1635 | 0.3209 | 0.5563 | 0.5292 | 0.1012 | 0.1989 | 0.5217 | 0.3871 | 0.0180^*^ |
| \| CHR = chromosome; SNP = single nucleotide polymorphism; EA = effect allele; RA = refractive astigmatism; J0_(RA)_ = J0 of refractive astigmatism; J45_(RA)_ = J45 of refractive astigmatism; IA = internal astigmatism; J0_(IA)_ = J0 of internal astigmatism; J45_(IA)_ = J45 of internal astigmatism; ADD = additive genetic model; DOM = dominant genetic model; REC = recessive genetic model  *P* values in bold with ** indicate study-wide significance level; ^*^ nominal significance level \| \| --- \| | | | | | | | | | | | | | | | | |

**Supplementary Figure 1.** Correlation matrix for keratometry readings, astigmatism and its vector components in children. K1, flat keratometry reading; K2, steep keratometry reading; CR, mean keratometry reading; CA, corneal astigmatism; J0_(CA)_, J0 of corneal astigmatism; J45_(CA)_, J45 of corneal astigmatism; RA, refractive astigmatism; J0_(RA)_, J0 of refractive astigmatism; J45_(RA)_, J45 of refractive astigmatism; IA, internal astigmatism; J0_(IA)_, J0 of internal astigmatism; J45_(IA)_, J45 of internal astigmatism. ***, *P* < 0.001; **, *P* < 0.01; *, *P* < 0.05. Minus signs indicate negative correlations.

**
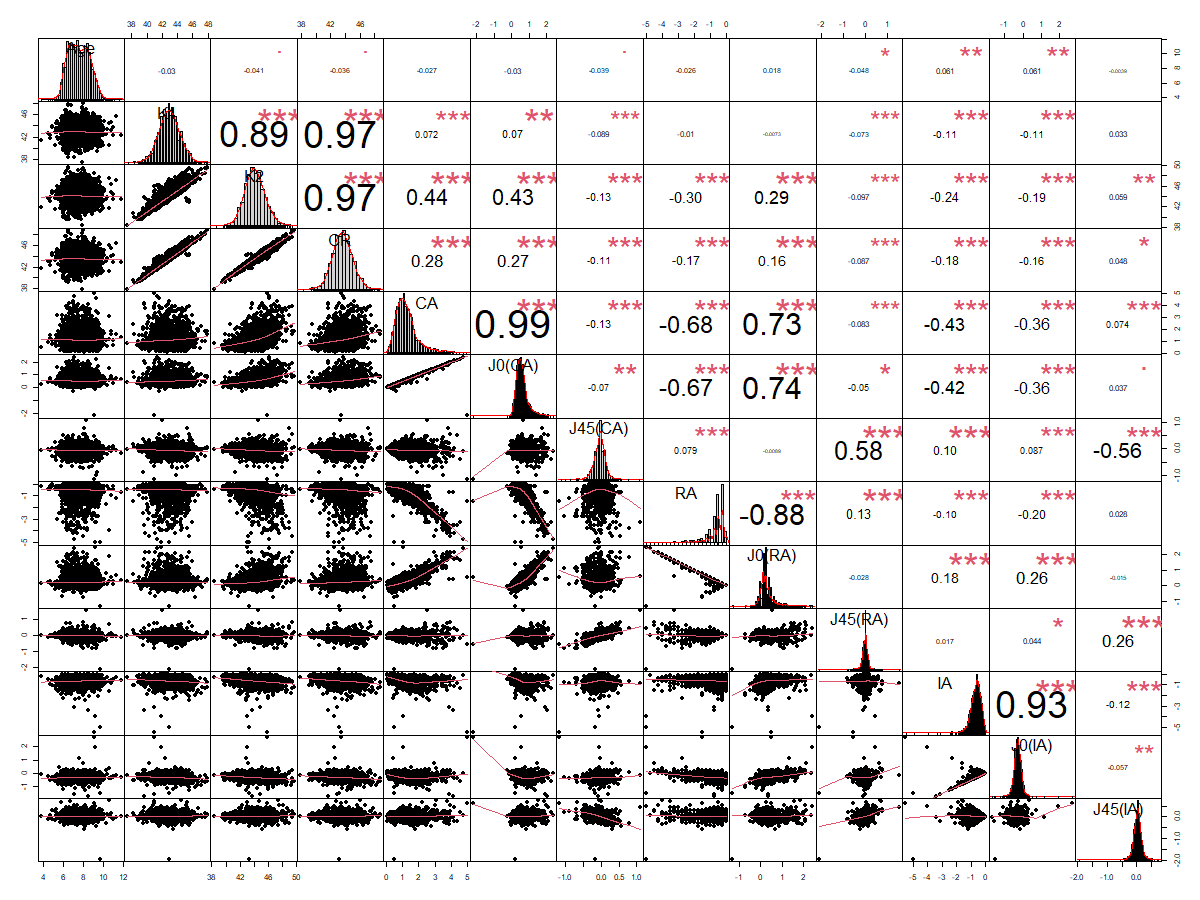
**

**Supplementary Figure 2.** Association of SNPs with components of corneal curvature, corneal astigmatism, refractive astigmatism, and internal astigmatism, stratified by sex. **a** Additive model, males; **b** Dominant model, males; **c** Recessive model, males; **d** Additive model, females; **e** Dominant model, females; **f** Recessive model, females. K1, flat keratometry reading; K2, steep keratometry reading; CR, mean keratometry reading; CA, corneal astigmatism; J0_(CA)_, J0 of corneal astigmatism; J45_(CA)_, J45 of corneal astigmatism; RA, refractive astigmatism; J0_(RA)_, J0 of refractive astigmatism; J45_(RA)_, J45 of refractive astigmatism; IA, internal astigmatism; J0_(IA)_, J0 of internal astigmatism; J45_(IA)_, J45 of internal astigmatism. The red horizontal line represents the study-wide significance threshold.


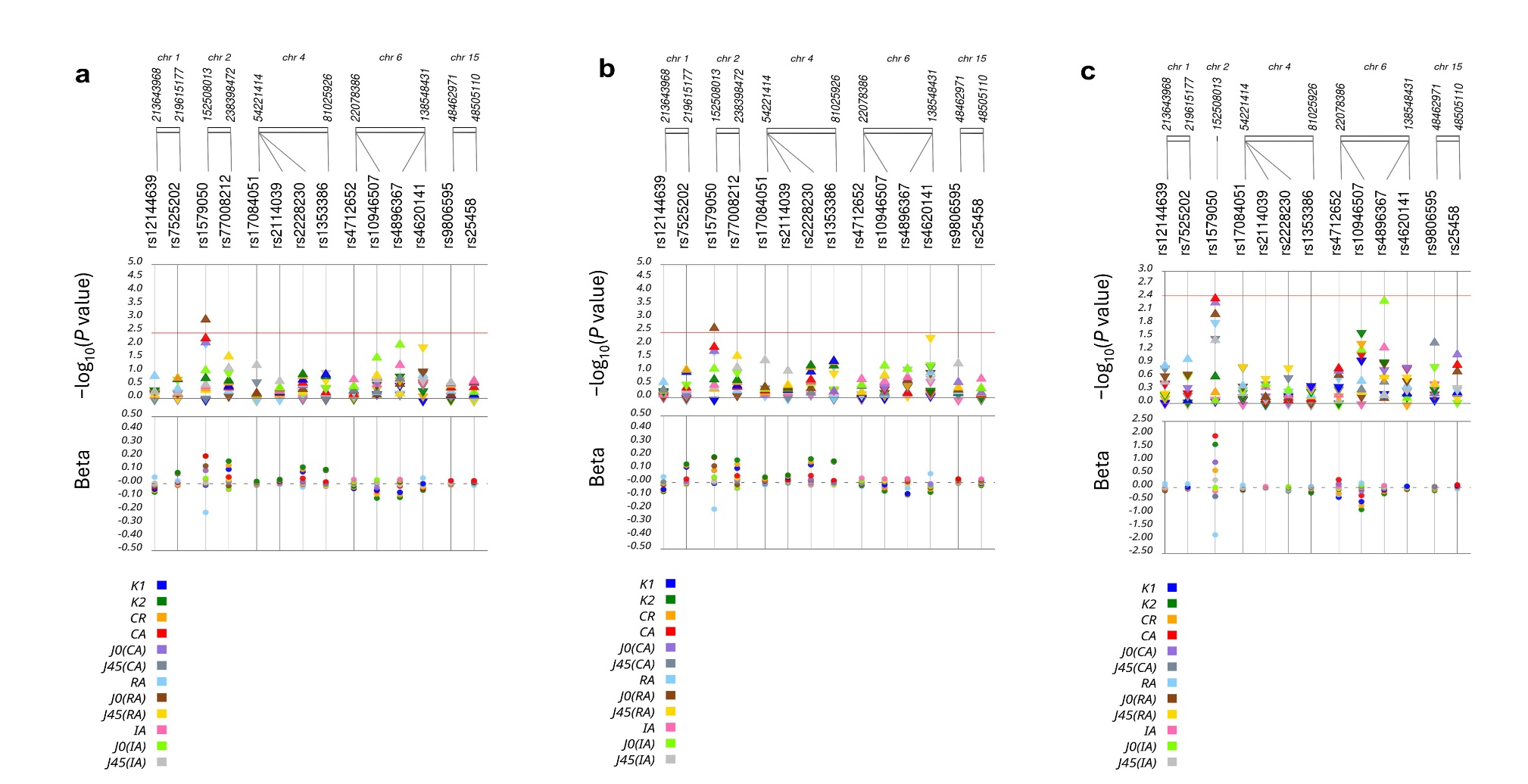


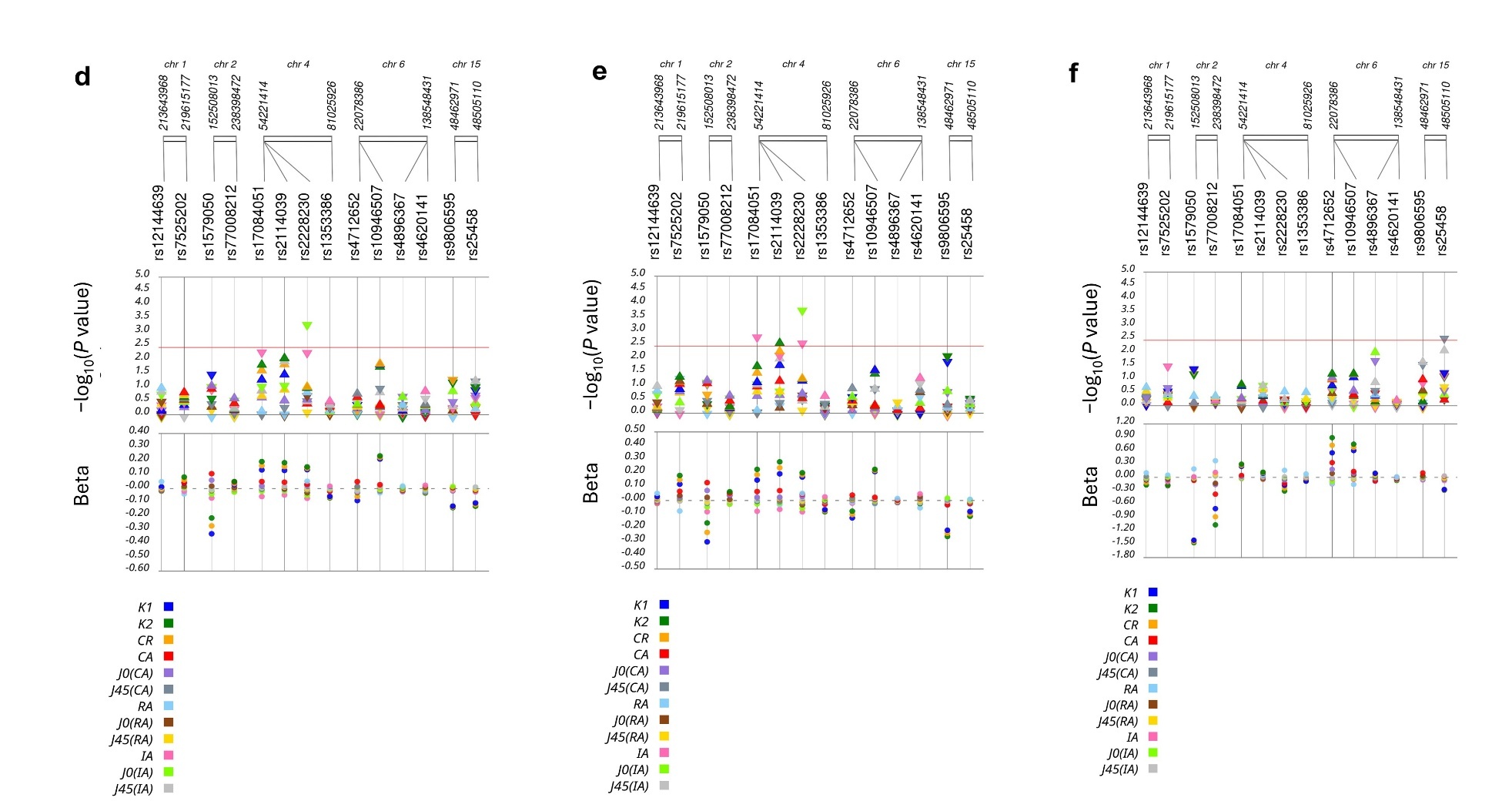


**Supplementary Figure 3.** Association of SNPs with components of corneal curvature, corneal astigmatism, refractive astigmatism, and internal astigmatism, stratified by age. **a** Additive model, 4- to 7-year-olds; **b** Dominant model, 4- to 7-year-olds; **c)** Recessive model, 4- to 7-year-olds; **d** Additive model, 8- to 11-year-olds; **e** Dominant model, 8- to 11-year-olds; **f** Recessive Model, 8- to 11-year-olds. K1, flat keratometry reading; K2, steep keratometry reading; CR, mean keratometry reading; CA, corneal astigmatism; J0_(CA)_, J0 of corneal astigmatism; J45_(CA)_, J45 of corneal astigmatism; RA, refractive astigmatism; J0_(RA)_, J0 of refractive astigmatism; J45_(RA)_, J45 of refractive astigmatism; IA, internal astigmatism; J0_(IA)_, J0 of internal astigmatism; J45_(IA)_, J45 of internal astigmatism. The red horizontal line represents the study-wide significance threshold.


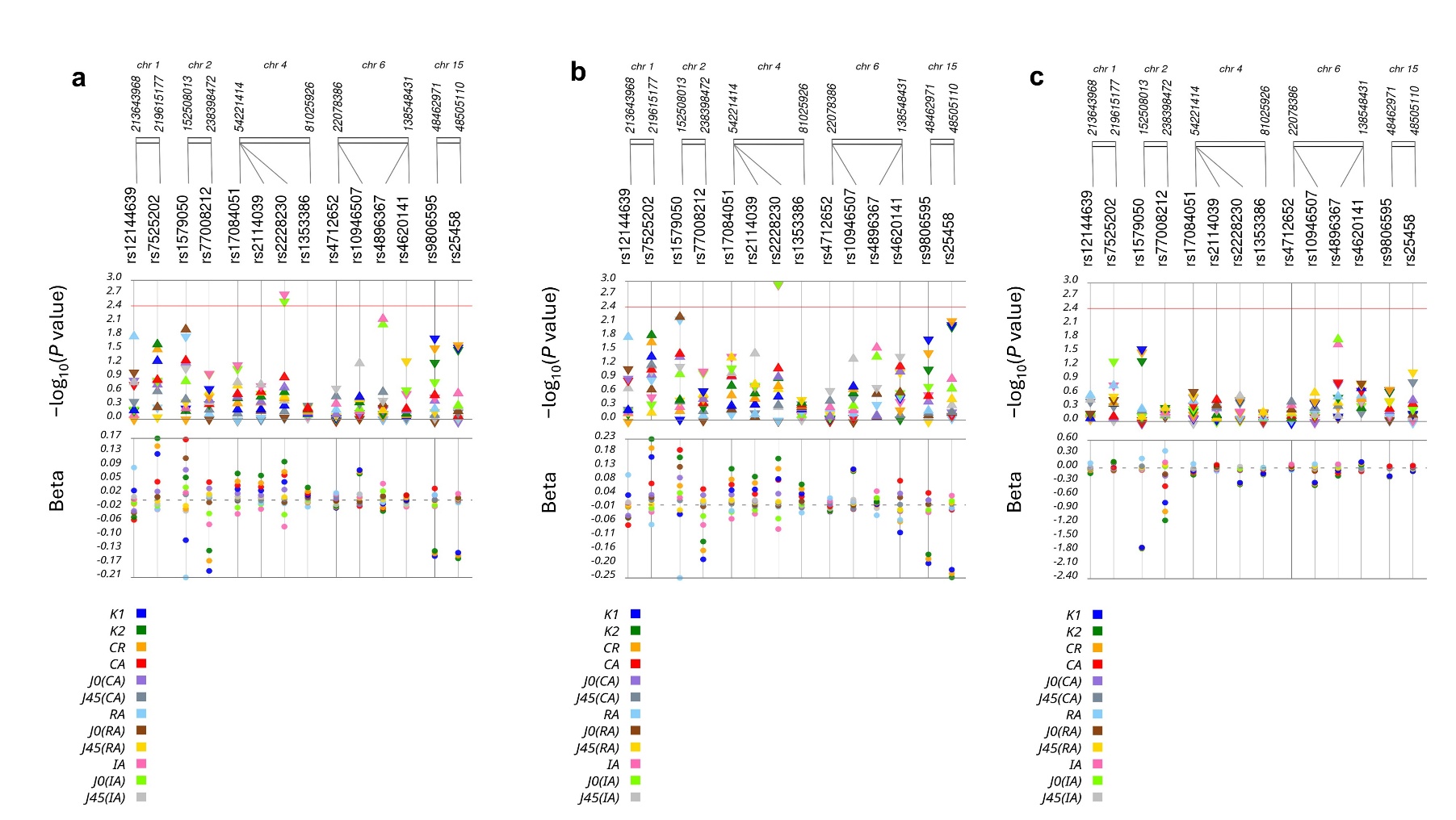


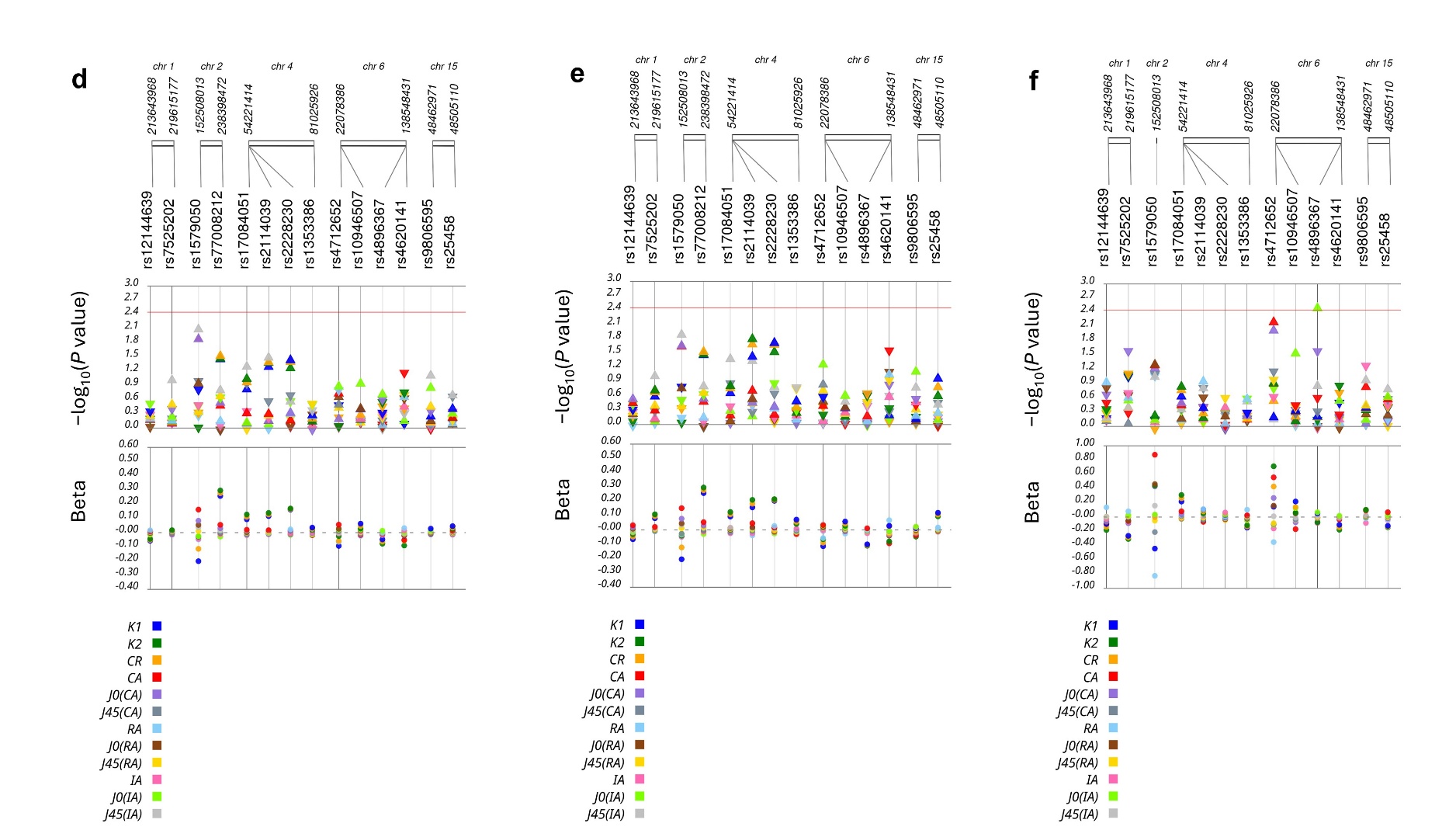


**Supplementary Figure 4.** Association of SNPs with dichotomous corneal, refractive, and internal astigmatism, stratified by sex. **a** Additive model, males; **b** Dominant model, males; **c** Recessive model, males; **d** Additive model, females; **e** Dominant model, females; **f** Recessive model, females. CA, corneal astigmatism; RA, refractive astigmatism; IA, internal astigmatism. The red horizontal line represents the study-wide significance threshold.


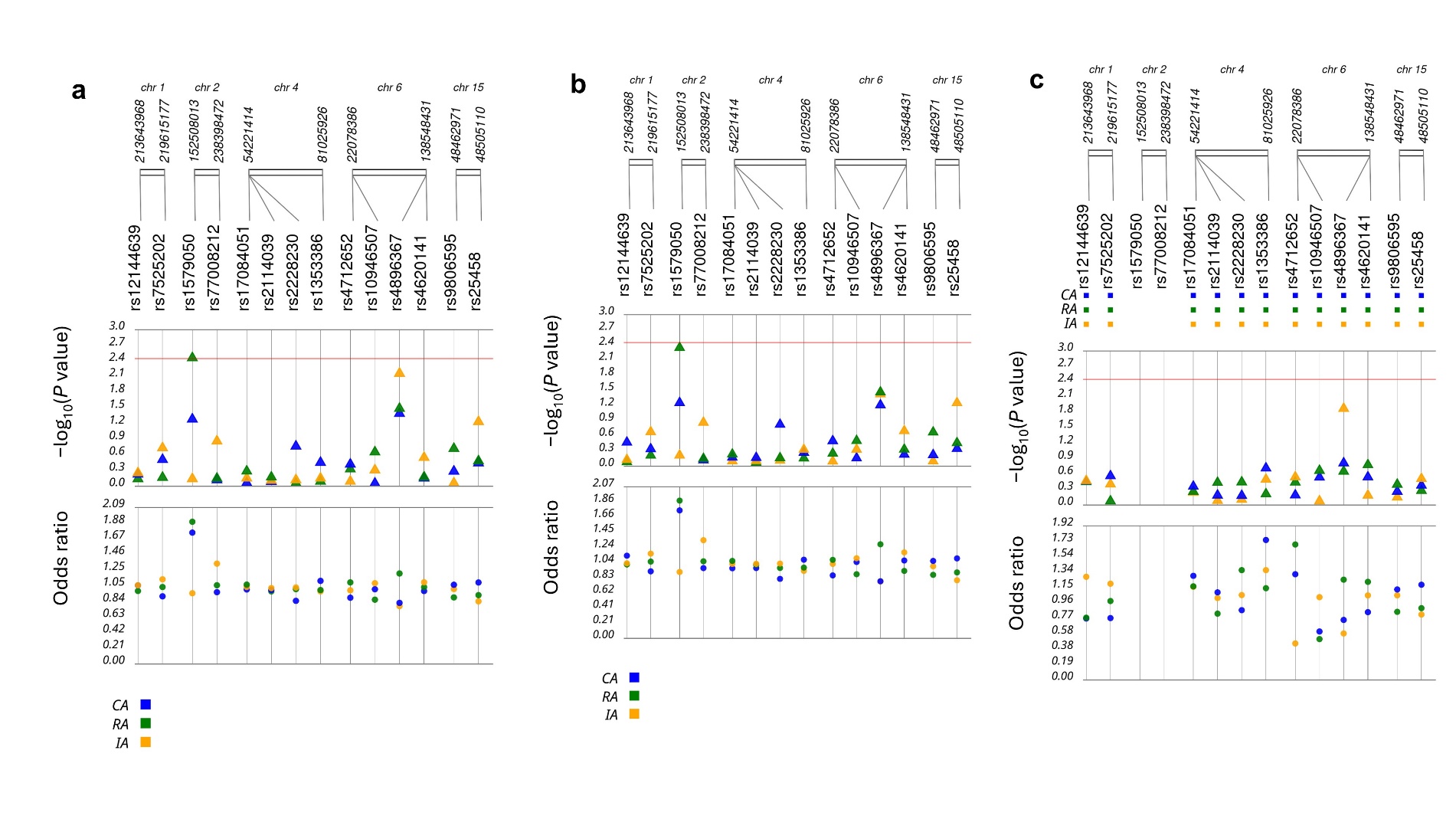


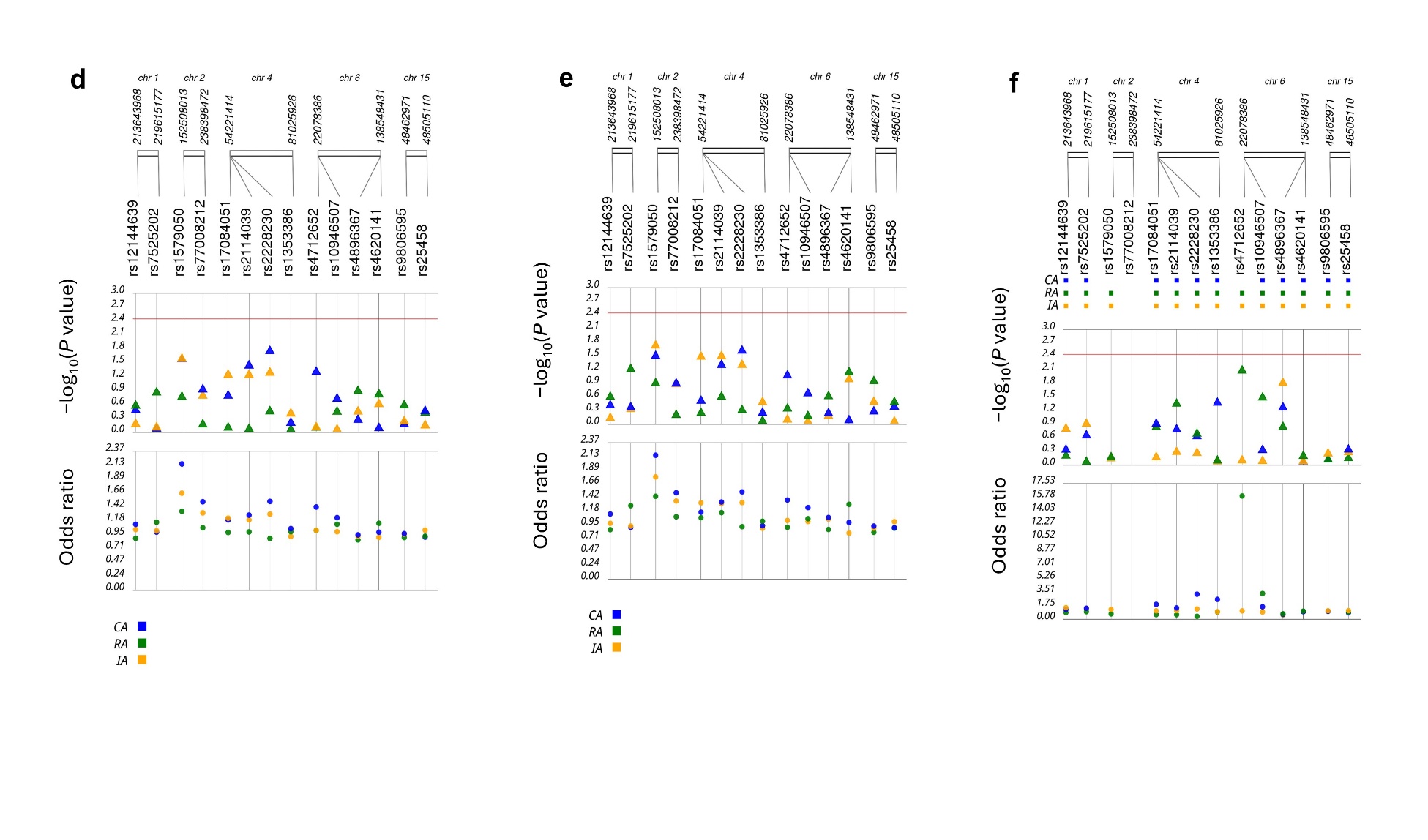


**Supplementary Figure 5.** Association of SNPs with dichotomous corneal, refractive, and internal astigmatism, stratified by age. **a** Additive model, 4- to 7-year-olds; **b** Dominant model, 4- to 7-year-olds; **c** Recessive model, 4- to 7-year-olds; **d** Additive model, 8- to 11-year-olds; **e** Dominant model, 8- to 11-year-olds; **f** Recessive model, 8- to 11-year-olds. CA, corneal astigmatism; RA, refractive astigmatism; IA, internal astigmatism. The red horizontal line represents the study-wide significance threshold.


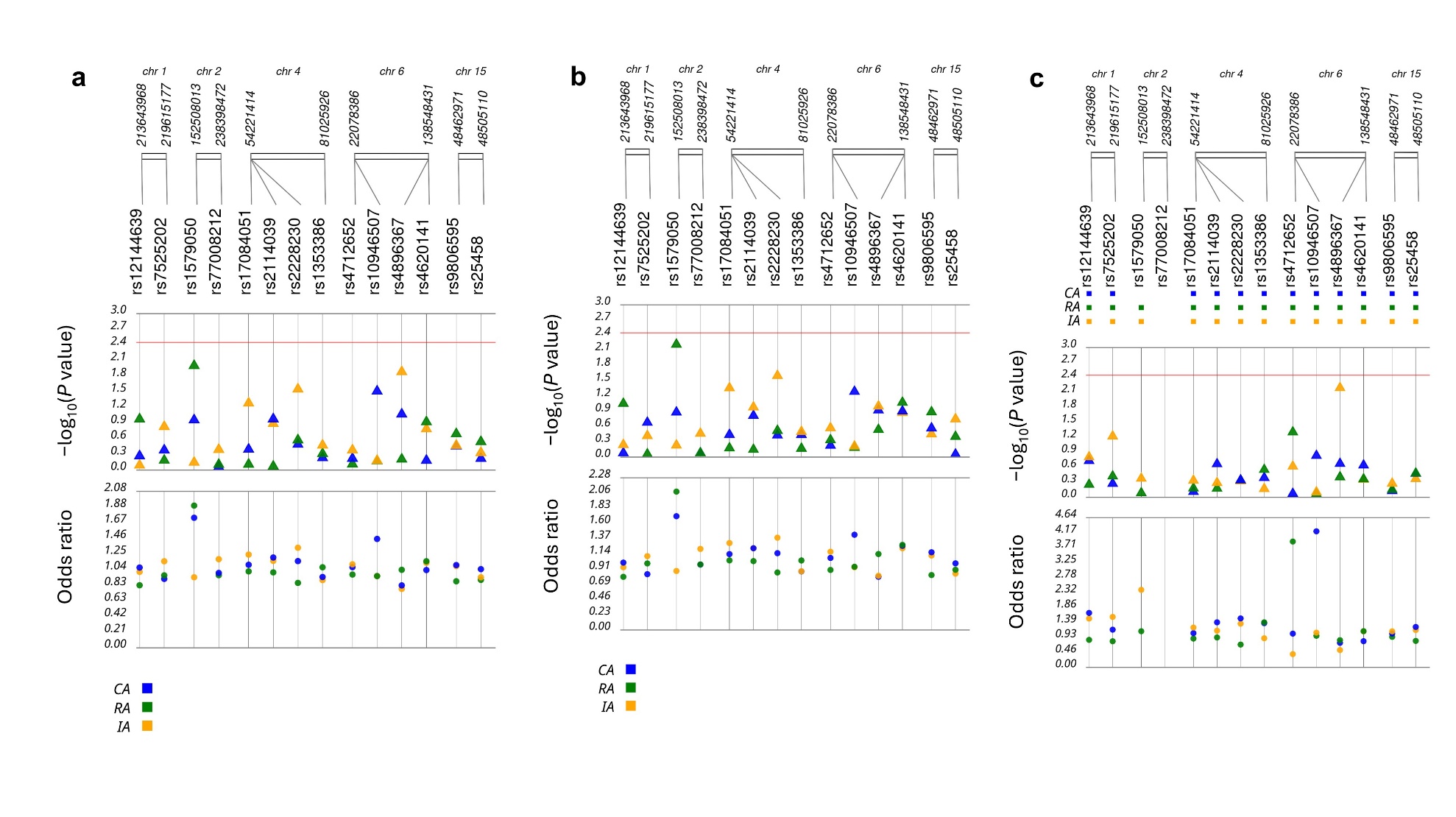


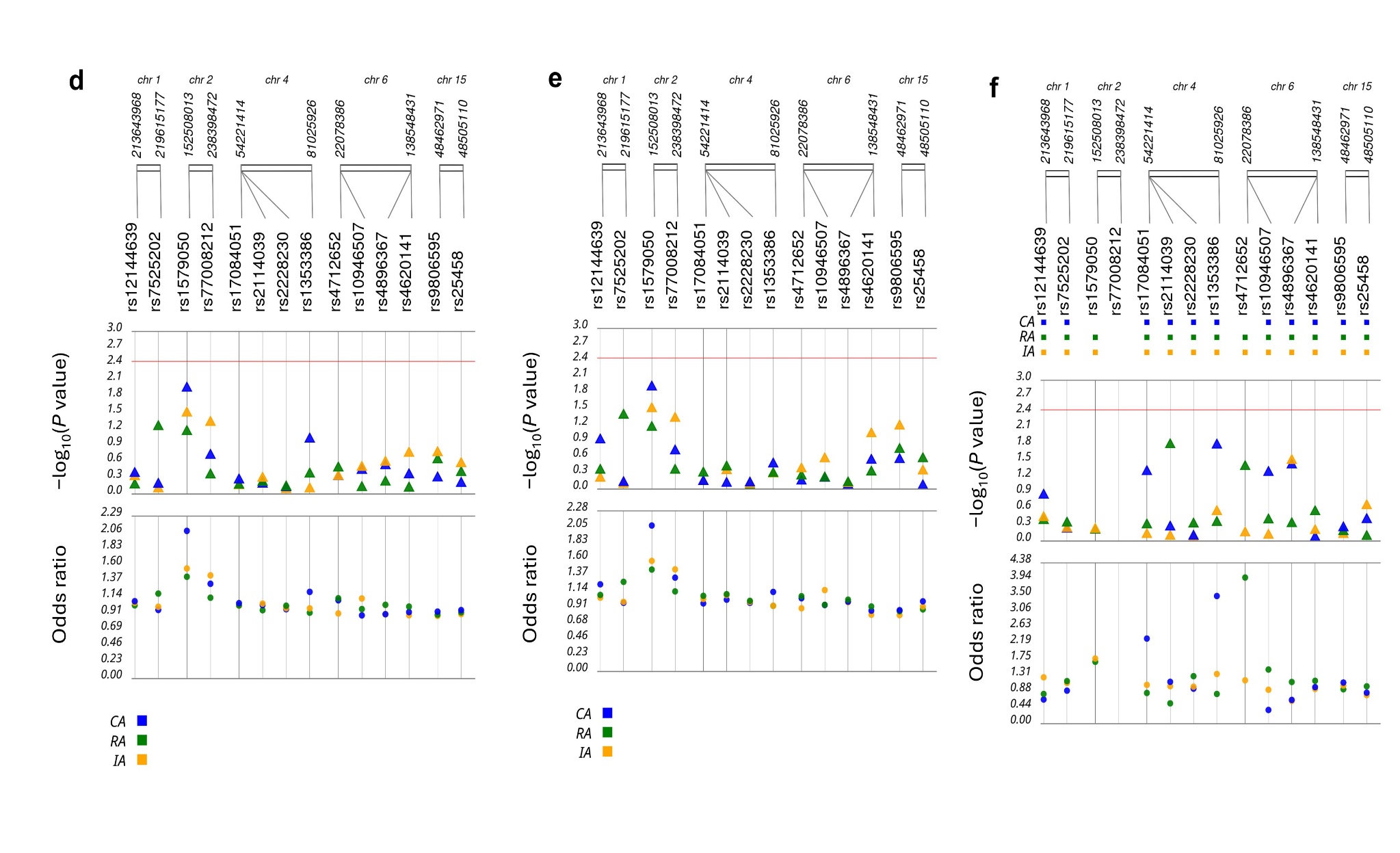


**References**

1. Shah RL, Li Q, Zhao W, Tedja MS, Tideman JWL, Khawaja AP, et al. A genome-wide association study of corneal astigmatism: The CREAM Consortium. Mol Vis. 2018;24:127-42.
2. Shah RL, Guggenheim JA. Genome-wide association studies for corneal and refractive astigmatism in UK Biobank demonstrate a shared role for myopia susceptibility loci. Hum Genet. 2018;137(11-12):881-96.
3. Guggenheim JA, McMahon G, Kemp JP, Akhtar S, St Pourcain B, Northstone K, et al. A genome-wide association study for corneal curvature identifies the platelet-derived growth factor receptor α gene as a quantitative trait locus for eye size in white Europeans. Mol Vis. 2013;19:243-53.
4. Han S, Chen P, Fan Q, Khor CC, Sim X, Tay WT, et al. Association of variants in FRAP1 and PDGFRA with corneal curvature in Asian populations from Singapore. Hum Mol Genet. 2011;20(18):3693-8.
5. Fan Q, Zhou X, Khor CC, Cheng CY, Goh LK, Sim X, et al. Genome-wide meta-analysis of five Asian cohorts identifies PDGFRA as a susceptibility locus for corneal astigmatism. PLoS Genet. 2011;7(12):e1002402.
6. Li Q, Wojciechowski R, Simpson C, Hysi P, Verhoeven V, Ikram M, et al. Genome-wide association study for refractive astigmatism reveals genetic co-determination with spherical equivalent refractive error: the CREAM consortium. Hum Genet. 2014;134.
7. Fan Q, Pozarickij A, Tan NYQ, Guo X, Verhoeven VJM, Vitart V, et al. Genome-wide association meta-analysis of corneal curvature identifies novel loci and shared genetic influences across axial length and refractive error. Communications Biology. 2020;3(1):133.
